# Outcomes of inflammatory bowel disease in patients with obesity following bariatric surgery: propensity score-matched cohort study

**DOI:** 10.1093/bjsopen/zraf086

**Published:** 2025-08-13

**Authors:** Erik Stenberg, Åsa H Everhov, Jonas Söderling, Johan Ottosson, Mehdi Osooli, SWIBREG study group, P Myrelid, H Strid, C Nordenvall, C Hedin, S Jäghult, J Halfvarson, O Grip, U L Fagerberg, K Mårild, Ellen Andersson, Daniel Bergemalm, Jonas F Ludvigsson, Carl Eriksson, Ola Olén

**Affiliations:** Department of Surgery, Faculty of Medicine and Health, Örebro University, Örebro, Sweden; Department of Clinical Science and Education, Södersjukhuset, Karolinska Institutet, Stockholm, Sweden; Department of Medical Epidemiology and Biostatistics, Karolinska Institutet, Solna, Sweden; Division of Clinical Epidemiology, Department of Medicine, Karolinska Institutet, Solna, Sweden; Division of Clinical Epidemiology, Department of Medicine, Karolinska Institutet, Solna, Sweden; Department of Surgery, Faculty of Medicine and Health, Örebro University, Örebro, Sweden; Division of Clinical Epidemiology, Department of Medicine, Karolinska Institutet, Solna, Sweden; Department of Biomedical and Clinical Sciences, Linköping University, Linköping, Sweden; Department of Surgery, Vrinnevi Hospital, Norrköping, Sweden; Department of Gastroenterology, Faculty of Medicine and Health, Örebro University, Örebro, Sweden; Department of Medical Epidemiology and Biostatistics, Karolinska Institutet, Solna, Sweden; Department of Pediatrics, Örebro University Hospital, Örebro, Sweden; Department of Medicine, Columbia University College of Physicians and Surgeons, New York, New York, USA; Division of Clinical Epidemiology, Department of Medicine, Karolinska Institutet, Solna, Sweden; Department of Gastroenterology, Faculty of Medicine and Health, Örebro University, Örebro, Sweden; Division of Clinical Epidemiology, Department of Medicine, Karolinska Institutet, Solna, Sweden; Department of Pediatric Gastroenterology and Nutrition, Sachs Children and Youth Hospital, Södersjukhuset, Stockholm, Sweden

## Abstract

**Background:**

Obesity is increasing among patients with inflammatory bowel disease, but bariatric surgery has been rare in this group owing to concerns about worsening the inflammatory bowel disease. The aim of the study was to evaluate inflammatory bowel disease-related outcomes following bariatric surgery.

**Methods:**

Nationwide cohort of all adult patients in Sweden between 2007 and 2020 with obesity and inflammatory bowel disease. Patients were matched 1 : 1 with a two-stage matching process between those undergoing bariatric surgery with those who did not (classified by inflammatory bowel disease subtype followed by a propensity score match including sex, age, number of previous targeted therapies, presence of immunotherapy, cumulative oral corticosteroid dose, and previous intestinal surgery). The primary composite outcome comprised inflammatory bowel disease-related hospitalization, initiation of corticosteroid therapy, immunomodulation, commencement of a new targeted therapy or major inflammatory bowel disease-related surgery.

**Results:**

The study included 798 patients with inflammatory bowel disease and obesity: 399 who underwent bariatric surgery (145 Crohn's disease, 238 ulcerative colitis, 16 unclassified inflammatory bowel disease) *versus* 399 who did not. Over a median observation period of 3.3 years in the surgery group and 3.0 years in the non-surgery group, the composite primary endpoint occurred in 201 patients who had surgery (incidence rate 11.9 (95% confidence interval (c.i.) 10.2 to 13.5) per 100 person-years) and 226 without surgery (incidence rate 15.1 (13.1 to 17.0) per 100 person-years), corresponding to an adjusted hazard ratio of 0.66 (95% c.i. 0.51 to 0.85) in those undergoing bariatric surgery compared with those who did not.

**Conclusion:**

Bariatric surgery was associated with improved inflammatory bowel disease-related outcomes among patients with inflammatory bowel disease and obesity, suggesting a potential benefit from bariatric surgery among patients with concomitant obesity and inflammatory bowel disease.

## Teaser

Bariatric surgery in patients with obesity and inflammatory bowel disease (IBD) remains controversial. In this matched cohort study of 399 patients who underwent bariatric surgery and 399 matched controls, bariatric surgery was associated with improved IBD-related outcomes suggesting a potential benefit from bariatric surgery among patients with concomitant obesity and IBD.

## Introduction

Both inflammatory bowel disease (IBD) and obesity are increasing, and up to 40% of patients with IBD may also have obesity^1^. Currently, bariatric surgery is the most effective treatment for severe obesity, with sleeve gastrectomy and Roux-en-Y gastric bypass being the two most widely used surgical methods^2,3^. Although the physiological mechanisms explaining the metabolic effects of sleeve gastrectomy and Roux-en-Y gastric bypass are not completely known, alteration of the gastrointestinal anatomy results in complex physiological changes, including modulation of gut hormones, alterations in bile flow, and changes to the intestinal microbiota^4^. Bariatric surgery can also reduce chronic inflammation^5^, and may therefore have an impact on existing IBD^6^. Although complication rates may be increased during bariatric surgery compared with those in patients without IBD, the rate of serious complications is considered acceptable for patients with IBD and severe obesity^7–9^. Small previous studies have reported improvement in IBD-related symptoms for patients with ulcerative colitis after bariatric surgery, whereas reports on the effect on patients with Crohn's disease have been conflicting (*Table S1*)^10–13^. In addition, an increased risk of new incident IBD following bariatric surgery has been suggested^14^. At present, concern about deterioration of IBD in general, and Crohn's disease in particular, has led to a situation in which many surgeons consider these diseases as relative contraindications to bariatric surgery^15^.

The aim of the present study was to compare IBD-related outcomes between patients with obesity and IBD with and without bariatric surgery (Roux-en-Y gastric bypass or sleeve gastrectomy).

## Methods

The study was approved by the Regional Ethical Review Board in Stockholm (2014/1287-31/4) and the Swedish Ethical Review Authority (2021-06209-01 with amendments: 2023-04868-02).

### Setting

This was a registry-based cohort study based on prospectively collected register data in Sweden. Healthcare in Sweden is tax-funded, providing equal access to all residents^16^. A unique personal identity number is assigned to all Swedish residents^17^, which enables linkage of nationwide registers. Data for the study population were retrieved from the Total Population Register (for migration, sex, birth date, death mortality)^18^, the Longitudinal Integrated Database for Health Insurance and Labour Market Studies (for information on education)^19^, the National Patient Register (for inpatient and outpatient hospital-based care)^20^, the Prescribed Drug Register (containing all prescriptions dispensed nationwide)^21^, ESPRESSO (Epidemiology Strengthened by histopathology Reports in Sweden, containing gastrointestinal histopathology data for all pathology registers nationwide)^22^, SWIBREG (Swedish IBD register, containing for example data on infusion biologicals and body mass index (BMI))^23,24^, and the Scandinavian Obesity Surgery Registry (SOReg)^25^ (*Table S2*).

### Study cohorts

#### Patients

**Fig. 1:**
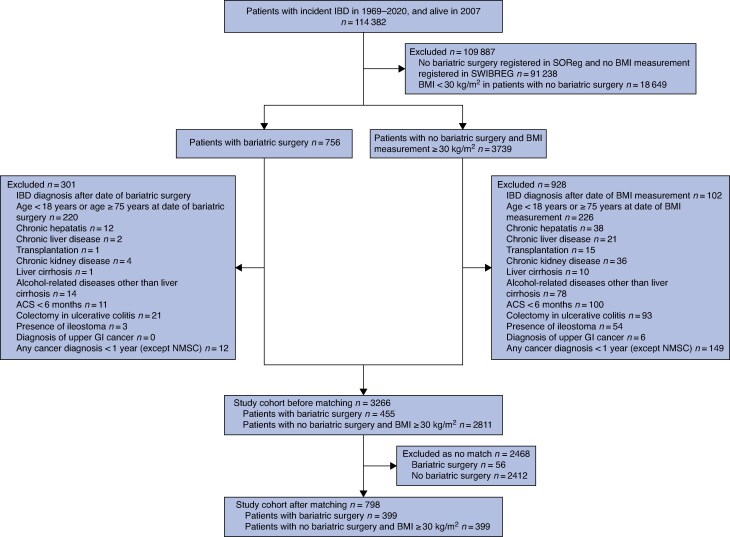
Study flow chart IBD, inflammatory bowel disease; SOReg, Scandinavian Obesity Surgery Registry; SWIBREG, Swedish IBD register; BMI, body mass index; ACS, acute coronary syndrome; GI, gastrointestinal; NMSC, non-melanoma skin cancer.

All Swedish patients were identified with an IBD diagnosis between 1969 and 2020 using validated register-based definitions of Crohn's disease, ulcerative colitis, or unclassified IBD^26–28^. The specific IBD diagnosis was determined from the latest two International Statistical Classification of Disease and Related Health Problems listings before bariatric surgery or matching. Eligible patients were adults (aged ≥ 18 but < 75 years), living in Sweden between 2007 and 2020. Exclusion criteria were chronic liver or kidney disease, alcohol-related liver disease, previous transplantation, colectomy (in patients with ulcerative colitis), presence of ileostomy, cancer diagnosis (except non-melanoma skin cancer) within 1 year or upper gastrointestinal cancer at any time, and acute coronary syndrome within 6 months (*Table S3* and *Fig. 1*). Patients were further classified by the presence of primary sclerosing cholangitis and previous IBD surgery (*Table S4–S7*).

#### Bariatric surgery cohort

The bariatric surgery cohort included patients with IBD and obesity (BMI ≥ 30 kg/m^2^) who underwent primary Roux-en-Y gastric bypass or sleeve gastrectomy between 2007 and 2020, registered in the SOReg^25^.

#### Non-bariatric surgery (control) cohort

The non-bariatric surgery cohort comprised matched patients with IBD and obesity (BMI ≥ 30 kg/m^2^), 2007 to 2020, who had not undergone bariatric surgery before or during the study period.

### Outcomes

The primary outcome was a composite measure of first occurrence of IBD-related hospitalization (IBD main diagnostic listing), start of systemic corticosteroid use, start of immunomodulatory therapy, start of a new targeted therapy (*Table S8*), or major IBD-related surgery (intestinal resection or stoma; *Table S7*) >30 days after the index date. Secondary outcomes were the above components considered separately: first IBD-related hospitalization; start of systemic corticosteroid use; start of immunomodulatory therapy; start of new therapy with immunomodulator or biological agent; or major IBD-related surgery (*Table S7 and S8*).

## Statistical analysis

Matching was done by sampling 1 : 1 without replacements in two steps: first, direct matching by IBD subtype followed by a propensity score-matching model, using a nearest function algorithm with a caliper of ≤ 0.2 including sex, age, and severity of IBD (number of previous types of targeted therapies (0, 1, ≥ 2), immunomodulator therapy (yes, no), cumulative oral corticosteroid dose in the past 2 years (0, 500–1500, 1501–3000, 3001–4500, > 4500 mg), and previous intestinal surgery (yes, no)). A standardized mean difference ≥ 0.10 was considered as residual imbalance between the groups and adjusted for in the statistical analyses. Day of surgery in the surgery group was considered to be index date. Owing to the large difference in BMI between groups, a sensitivity analysis including BMI as a variable in the propensity score match was included as an amendment to the original study plan. Postoperative complications were graded according to the Clavien–Dindo classification; a complication defined as Clavien–Dindo grade ≥ I was considered a postoperative complication^29^.

The definition and sources of the co-variables included are presented in detail in *Tables S2–S11*. Continuous variables are presented as mean(standard deviation, s.d.); those not assuming a normal distribution are presented as median (interquartile range). Categorical variables are presented as numbers with percentages. Outcomes are presented as incidence rates with 95% confidence intervals, using Cox regression with hazard ratios (HRs) and 95% confidence intervals as measures of association. After testing the proportional hazards assumption, Cox regression analysis was conducted in two models, the first conditioned on the matching set (unadjusted model), and the second adjusted for previous intestinal surgery, ischaemic heart disease, congestive heart failure, hypertension, and any form of diabetes (adjusted model). Time-dependent outcomes were estimated and visualized using the Kaplan–Meier method and presented as a cumulative probability (1 – Kaplan–Meier estimate). For these analyses, patients were followed until the first incidence of the outcome and censored by emigration, death, or on 31 December 2021, whichever came first.

Statistical analyses were conducted using SAS^®^ version 9.4 (SAS Institute, Cary, NC, USA) and Stata^®^ version 17.0 (StataCorp, College Station, TX, USA).

## Results

During the study period, 114 382 adults with IBD who were living in Sweden in 2007 were identified. After exclusion of 109 887 with missing information on BMI (91 238) or with a BMI < 30 kg/m^2^ (18 649), and 301 patients in the surgery group and 928 in the non-surgery group who had at least 1 exclusion criterion, 455 patients who underwent bariatric surgery and 2811 patients with obesity who did not undergo bariatric surgery were available for matching (*Fig. 1*). Patients in the surgery group were more often women, younger, with a higher BMI, and had more metabolic co-morbidities. A higher proportion had been born in a Nordic country, had a higher level of education, and a lower severity of IBD disease than those who did not undergo bariatric surgery (*Table S12*). The propensity score match generated two well matched groups with residual imbalance for previous intestinal surgery. Patients in the surgery group had higher BMI and more often had associated metabolic co-morbidities (*Table 1*).

**Table 1:** Baseline characteristics of patients after matching

	Surgery group (*n* = 399)	Control group (*n* = 399)	Standardized mean difference
Age (years), mean(s.d.)	33.5(10.7)	33.0(10.7)	0.043
**Sex**			
Female	316 (79.2%)	314 (78.7%)	0.012
Male	83 (20.8%)	85 (21.3%)	0.012
Body mass index (kg/m^2^), mean(s.d.)	40.6(5.3)	34.0(5.5)	1.231
**Co-morbidities**			
Ischaemic heart disease	6 (1.5%)	2 (0.5%)	0.101
Congestive heart failure	7 (1.8%)	2 (0.5%)	0.119
Hypertension	168 (42.1%)	94 (23.6%)	0.403
Diabetes mellitus	75 (18.8%)	31 (7.8%)	0.329
**IBD diagnosis**			
Crohn's disease	145 (36.3%)	145 (36.3%)	0.000
Ulcerative colitis	238 (59.6%)	238 (59.6%)	0.000
Unclassified IBD	16 (4.0%)	16 (4.0%)	0.000
Disease duration at index date (years), mean(s.d.)	10.3(8.1)	10.9(7.4)	0.070
**Previous types of targeted therapies**	356 (89.2%)	364 (91.2%)	0.068
0	34 (8.5%)	27 (6.8%)	0.066
1	9 (2.3%)	8 (2.0%)	0.017
≥ 2	356 (89.2%)	364 (91.2%)	0.068
Any immunomodulator therapy before index date	139 (34.8%)	137 (34.3%)	0.011
Cumulative oral corticosteroids within 2 years before index date (mg prednisolone equivalents), mean(s.d.)	294(74)	298(75)	0.023
Any intestinal surgery before index date	27 (6.8%)	39 (9.8%)	0.109
**Extraintestinal manifestations at start of follow-up**			
Primary sclerosing cholangitis	3 (0.8%)	11 (2.8%)	0.153
Other	81 (20.3%)	65 (16.3%)	0.104
**Education level (years)**			
Primary (≤ 9 years)	42 (10.5%)	47 (11.8%)	0.040
Secondary (10–12 years)	251 (62.9%)	220 (55.1%)	0.158
Higher (> 12 years)	106 (26.6%)	131 (32.8%)	0.137
Missing	0 (0%)	1 (0.3%)	0.071
**Country of birth**			
Nordic	372 (93.2%)	361 (90.5%)	0.101
Non-Nordic	25 (6.3%)	32 (8.0%)	0.068
Missing	2 (0.5%)	6 (1.5%)	0.101

Values are *n* (%) unless otherwise stated. s.d., Standard deviation. IBD, inflammatory bowel disease.

In the surgery group, 191 patients underwent Roux-en-Y gastric bypass (47.9%) and 208 had sleeve gastrectomy (52.1%). Mean(s.d.) absolute weight loss at 1 year after surgery was 36(10) kg (total weight loss 31(7)%) after RYGB, and 31(11) kg (total weight loss 27(8)%) after SG.

### Postoperative complications

A postoperative complication occurred during the first 30 days after bariatric surgery in 32 patients (8.0%). The most common complication registered was leak or intra-abdominal abscess/infection, which occurred in nine patients (2.3%), followed by bleeding in six (1.5%) (*Table S13*). Patients who developed a postoperative complication had a similar rate of occurrence of the composite endpoint (50.0%; incidence rate 9.2 (95% confidence interval (c.i.) 4.7 to 13.7) per 100 person-years) as patients who did not have a postoperative complication (50.4%; incidence rate 12.2 (10.4 to 13.9) per 100 person-years).

### IBD-related outcome

**Fig. 2:**
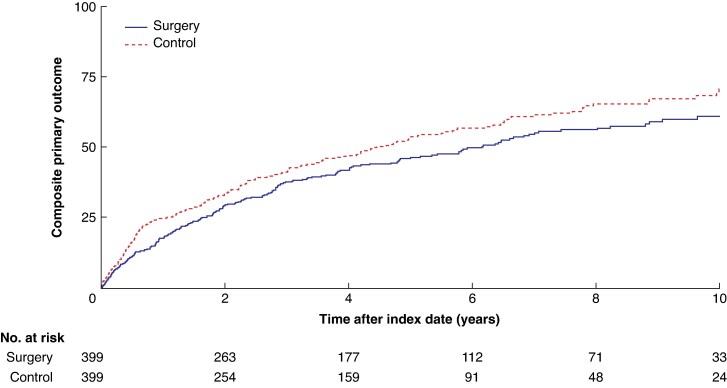
Kaplan–Meier failure curve for time to composite primary outcome The primary outcome included first occurrence of inflammatory bowel disease (IBD)-related hospitalization, start of systemic corticosteroid use, immunomodulatory therapy or new target therapy, or major IBD-related surgery.

Over a median observation period of 3.3 years (IQR 1.4 to 6.5) in the surgery group and 3.0 years (IQR 1.1 to 5.7) in the non-surgery group, the composite primary endpoint occurred in 201 patients who had bariatric surgery (50.4%; incidence rate 11.9 (95% c.i. 10.2 to 13.5) per 100 person-years) and 226 patients in the no-surgery group (56.6%; incidence rate 15.1 (13.1 to 17.0) per 100 person-years) (adjusted HR 0.66, 95% c.i. 0.51 to 0.85) (*Fig. 2*). The HR for surgery *versus* no surgery was significantly lower for both patients with Crohn's disease (adjusted HR 0.63, 0.42 to 0.96) and those with ulcerative colitis (adjusted HR 0.64, 0.45 to 0.92). When stratified by type of bariatric surgery, similar patterns were seen, although statistical significance was reached after Roux-en-Y gastric bypass (unadjusted HR 0.73, 0.57 to 0.92; adjusted HR 0.53, 0.35 to 0.80), but not after sleeve gastrectomy after adjustment (unadjusted HR 0.73, 0.58 to 0.92; adjusted HR 0.73, 0.52 to 1.03).

For the secondary endpoints, there was a statistically significantly lower risk of IBD-related hospitalization and systemic corticosteroid use among patients with ulcerative colitis, and a lower risk of starting targeted therapy for the overall IBD group, after surgery compared with the no-surgery group (*Table 2*, *Figs. S14 and S15*).

**Table 2:** Risk of IBD-related hospitalization, first systemic corticosteroid use, and first major IBD-related surgery in patients who had bariatric surgery *versus* matched controls

	No. of patients†	No. of events		Incidence rate per 100 person-years*		Hazard ratio*	
		Surgery	Control	Surgery	Control	Unadjusted‡	Adjusted§
**Primary outcome**							
IBD subtype							
IBD overall	399 (100%)	201 (50.4%)	226 (56.6%)	11.9 (10.2, 13.5)	15.1 (13.1, 17.0)	0.73 (0.62, 0.86)	0.66 (0.51, 0.85)
CD	145 (36.3%)	84 (57.9%)	91 (62.8%)	15.6 (12.2, 18.9)	20.0 (15.9, 24.1)	0.69 (0.52, 0.90)	0.63 (0.42, 0.96)
UC	238 (59.6%)	112 (47.1%)	128 (53.8%)	10.1 (8.2, 12.0)	12.7 (10.5, 14.9)	0.80 (0.64, 0.99)	0.64 (0.45, 0.92)
Unclassified IBDU	16 (4.0%)	5 (31.3%)	7 (43.8%)	11.2 (1.4, 21.0)	17.6 (4.6, 30.6)	0.17 (0.03, 1.05)	0.33 (0.03, 3.20)
Type of surgery							
RYGB	191 (47.9%)	94 (49.2%)	114 (59.7%)	9.4 (7.5, 11.2)	13.1 (10.7, 15.5)	0.73 (0.57, 0.92)	0.53 (0.35, 0.80)
SG	208 (52.1%)	107 (51.4%)	112 (53.8%)	15.5 (12.6, 18.5)	17.8 (14.5, 21.2)	0.73 (0.58, 0.92)	0.73 (0.52, 1.03)
**Secondary outcomes**							
IBD-related hospitalization							
IBD overall	399 (100%)	37 (9.3%)	46 (11.5%)	1.4 (1.0, 1.9)	1.8 (1.3, 2.3)	0.78 (0.56, 1.08)	0.70 (0.41, 1.16)
CD	145 (36.3%)	18 (12.4%)	17 (11.7%)	2.1 (1.1, 3.1)	2.0 (1.0, 2.9)	1.07 (0.65, 1.76)	0.87 (0.38, 1.97)
UC	238 (59.6%)	16 (6.7%)	27 (11.3%)	1.0 (0.5, 1.4)	1.7 (1.0, 2.3)	0.56 (0.35, 0.91)	0.45 (0.20, 0.98)
Systemic corticosteroid use							
IBD overall	399 (100%)	158 (39.6%)	178 (44.6%)	8.2 (6.9, 9.5)	10.0 (8.5, 11.5)	0.77 (0.64, 0.92)	0.70 (0.53, 0.93)
CD	145 (36.3%)	64 (44.1%)	64 (44.1%)	10.0 (7.5, 12.4)	10.4 (7.8, 12.9)	0.82 (0.61, 1.10)	0.77 (0.49, 1.22)
UC	238 (59.6%)	91 (38.2%)	108 (45.4%)	7.3 (5.8, 8.9)	9.7 (7.8, 11.5)	0.77 (0.61, 0.97)	0.62 (0.42, 0.90)
Start of immunomodulatory therapy							
IBD overall	399 (100%)	82 (20.6%)	95 (23.8%)	3.5 (2.8, 4.3)	4.3 (3.4, 5.1)	0.80 (0.63, 1.01)	0.78 (0.55, 1.12)
CD	145 (36.3%)	38 (26.2%)	42 (29.0%)	4.9 (3.4, 6.5)	5.8 (4.1, 7.6)	0.75 (0.53, 1.07)	0.76 (0.44, 1.31)
UC	238 (59.6%)	40 (16.8%)	51 (21.4%)	2.6 (1.8, 3.5)	3.5 (2.6, 4.5)	0.79 (0.57, 1.09)	0.65 (0.38, 1.11)
Start of new targeted therapy							
IBD overall	399 (100%)	46 (11.5%)	59 (14.8%)	1.8 (1.3, 2.3)	2.3 (1.7, 2.9)	0.70 (0.52, 0.94)	0.60 (0.37, 0.96)
CD	145 (36.3%)	28 (19.3%)	27 (18.6%)	3.3 (2.1, 4.6)	3.2 (2.0, 4.3)	0.96 (0.63, 1.45)	0.82 (0.42, 1.59)
UC	238 (59.6%)	16 (6.7%)	28 (11.8%)	1.0 (0.5, 1.4)	1.7 (1.1, 2.3)	0.54 (0.33, 0.87)	0.57 (0.27, 1.21)
Major IBD-related surgery							
IBD overall	399 (100%)	14 (3.5%)	22 (5.5%)	0.5 (0.2, 0.8)	0.8 (0.5, 1.2)	0.67 (0.39, 1.13)	0.84 (0.36, 1.96)
CD	145 (36.3%)	8 (5.5%)	8 (5.5%)	0.9 (0.3, 1.5)	0.9 (0.3, 1.5)	0.86 (0.40, 1.86)	0.62 (0.15, 2.64)
UC	238 (59.6%)	5 (2.1%)	13 (5.5%)	0.3 (0, 0.5)	0.8 (0.4, 1.2)	0.45 (0.20, 1.01)	0.57 (0.17, 1.95)

Values are *n* (%) unless otherwise stated. *Values in parentheses are 95% confidence intervals. †Numbers were similar for bariatric surgery and control groups. IBD, inflammatory bowel disease; CD, Crohn's disease; UC, ulcerative colitis; RYGB, Roux-en-Y gastric bypass; SG, sleeve gastrectomy. ‡Conditioned on matching set;. §conditioned on matching set and further adjusted for previous intestinal surgery, ischaemic heart disease, congestive heart failure, hypertension, and diabetes.

### Sensitivity analysis

Including exact BMI in a second matching resulted in 221 patients in the surgery group and 221 matched patients who did not undergo bariatric surgery. During observation, 106 patients (48.0%) in the surgery group and 118 controls (53.4%) reached the composite endpoint (unadjusted HR 0.72, 95% c.i. 0.57 to 0.91; adjusted HR 0.72, 0.50 to 1.02) (*Table S16*, *Fig. S17*, *Table S18*).

## Discussion

Bariatric surgery was associated with a lower risk of worsening IBD. The risk was reduced both in ulcerative colitis and in Crohn's disease. A similar tendency was seen after sleeve gastrectomy as well as Roux-en-Y gastric bypass, but the results were statistically significant only after Roux-en-Y gastric bypass surgery.

The findings confirm the results of earlier smaller studies^10–13^ suggesting an improvement after bariatric surgery for patients with ulcerative colitis. In the present study, bariatric surgery was also associated with reduced severity of disease for patients with Crohn's disease. A French study including 326 patients with Crohn's disease and 261 patients with ulcerative colitis who had mainly undergone sleeve gastrectomy reported increased readmission rates over 2 years of follow-up among patients with Crohn's disease^9^. However, owing to lack of comparison with a matched non-operated control group, the results of that study may be explained by generally higher readmission rates among patients with Crohn's disease compared with patients with ulcerative colitis, irrespective of bariatric surgery^30^. During follow-up in the present study, patients with ulcerative colitis experienced a lower risk of IBD-related hospitalization, and the overall IBD group had reduced use of systemic corticosteroids and start of targeted therapy. No statistically significant differences were observed between groups for other outcomes, possibly owing to limited power.

The results suggest no worsening and potentially an improvement in IBD treatment outcomes after bariatric surgery. The association between bariatric surgery and IBD outcomes is likely multifactorial and complex, including a combination of reduced general inflammation and altered intestinal microbiota after bariatric surgery. A recent study^31^ reported reduced DNA methylation of cells in the rectal mucosa after Roux-en-Y gastric bypass, suggesting reduced inflammation of the bowel wall. Sleeve gastrectomy has been reported to reduce inflammation of the bowel, potentially through a mechanism related to the microbiota^32^. In a previous comparison of the two surgical methods^33^, a greater reduction in general inflammation was observed after Roux-en-Y gastric bypass compared with sleeve gastrectomy. Although weight-independent effects may contribute, greater effectiveness in terms of long-term weight loss and greater efficacy of drugs in patients with lower BMI may account for a large proportion of the differences^34,35^.

The present and previous^6,9^ studies have reported that bariatric surgery can be performed among patients with IBD with acceptable perioperative and postoperative safety, and reasonable postoperative weight loss. The results of this study, with more pronounced effects after Roux-en-Y gastric bypass, contradict a report of better IBD outcomes after sleeve gastrectomy in a recent study^36^ (including 154 patients who underwent Roux-en-Y gastric bypass and 328 after sleeve gastrectomy) for a subgroup of a large population. As complication rates have been reported to be higher after Roux-en-Y gastric bypass, in particular for patients with ulcerative colitis, sleeve gastrectomy may be preferable from a safety perspective^6,8^. However, considering the better long-term weight loss and metabolic effects of Roux-en-Y gastric bypass and the high incidence of gastro-oesophageal reflux disease after sleeve gastrectomy^37^, more studies are needed on the perioperative safety of Roux-en-Y gastric bypass in patients with IBD. There is a particular need for studies with a focus on subgroups that may undergo surgery safely to clarify who may benefit most from each surgical procedure, not least given the association with IBD improvements seen in the present study.

Weight loss can also be achieved using modern pharmacological treatment such as glucagon-like peptide-1 receptor agonist treatment, a treatment that has also been associated with improved IBD outcomes in patients with obesity^38^. Historically, these drugs have not been widely accessible for obesity management in Sweden, mainly as they are not subsidized for obesity treatment. Comparative studies are needed to better understand the risks and benefits of bariatric surgery and modern pharmacological obesity management in patients with obesity and IBD.

Major strengths of the present study were the nationwide coverage, high quality of data, and matched design. However, the results must be viewed in light of the limitations. Despite the matched study design, the study remains non-randomized; as such, residual imbalance remains between the groups, and so any causal interpretations must be made with caution. Furthermore, because of missing information on BMI for many patients in the non-operated group, this variable could not be included in the propensity score matching. Obesity has been reported to have only minor negative effects on IBD disease progression, but may reduce the efficacy of pharmacological treatment^39^. Thus, the higher BMI of the surgical group in the present study may potentially have led to underestimation of the true treatment effects of bariatric surgery. However, a sensitivity analysis including BMI in the propensity score matching yielded similar results (*Fig. S17*). The present study was conducted in a Scandinavian country, including a mainly white, northern European population with a high incidence of IBD^40^. The gaps in IBD incidence between different geographical regions have been reported to narrow in more recent years and are thus unlikely to have influenced the relevance to other populations^41^.

## Collaborators

Members of the SWIBREG study group: P. Myrelid (Linköping University, Linköping, Sweden); H. Strid, C. Nordenvall, C. Hedin (Karolinska Institutet, Stockholm Sweden and Karolinska University Hospital, Stockholm, Sweden); S. Jäghult (Södersjukhuset, Stockholm, Sweden); J. Halfvarson (Örebro University, Örebro, Sweden); O. Grip (Skåne University Hospital, Malmö, Sweden); U. L. Fagerberg (Karolinska Institutet, Stockholm, Sweden and Region Vastmanland, Vasterås, Sweden); K. Mårild (Sahlgrenska Academy, Gothenburg, Sweden and Queen Silvia Children's Hospital, Gothenburg, Sweden).

## Supplementary Material

zraf086_Supplementary_Data

## Data Availability

Data cannot be shared publicly because of patient confidentiality under current Swedish legislation. Data are available from SOReg (contact via soreg@regionorebrolan.se), the Swedish Board of Health and Welfare (contact via Registerservice@socialstyrelsen.se), and Statistics Sweden (contact via mikrodata@scb.se) for researchers who meet the criteria for access to confidential data.
