# Supplementary material for: Outcomes of inflammatory bowel disease in patients with obesity following bariatric surgery: propensity score-matched cohort study
Source: BJS Open. 2025 Aug 13;9(4):zraf086. doi: 10.1093/bjsopen/zraf086 (PMC12345414; doi:10.1093/bjsopen/zraf086)
Supplement: zraf086_Supplementary_Data [file zraf086_supplementary_data.docx]

**Outcomes of inflammatory bowel disease in patients with obesity following bariatric surgery – propensity score matched cohort study**

Erik Stenberg^1^, Åsa H Everhov^2, 3, 4^, Jonas Söderling^4^, Johan Ottosson^1^, Mehdi Osooli^4^, the SWIBREG study group*^§^*, Ellen Andersson^5,6^, Daniel Bergemalm^7^, Jonas F Ludvigsson^3,8,9^, Carl Eriksson^4,7^, Ola Olén^4,10^

^1^Department of Surgery, Faculty of Medicine and Health, Örebro University, Örebro, Sweden

^2^Department of Clinical Science and Education, Södersjukhuset, Karolinska Institutet, Stockholm, Sweden

^3^Department of Medical Epidemiology and Biostatistics, Karolinska Institutet, Solna, Sweden

^4^Division of Clinical Epidemiology, Department of Medicine, Karolinska Institutetet, Solna, Sweden

^5^Department of Biomedical and Clinical Sciences, Linköping University, Linköping, Sweden

^6^Department of Surgery, Vrinnevi Hospital, Norrköping, Sweden

^7^Department of Gastroenterology, Faculty of Medicine and Health, Örebro University, Örebro, Sweden

^8^Department of Pediatrics, Örebro University Hospital, Sweden

^9^Department of Medicine, Columbia University College of Physicians and Surgeons, New York, NY, USA

^10^Sachs Children and Youth Hospital, Södersjukhuset, Stockholm, Sweden

**Corresponding author.** Erik Stenberg, Department of Surgery, Örebro University Hospital, 70182 Örebro, Sweden. **ORCID ID 0000-0001-9189-0093**; **Twitter: ErikStenberg_MD**

**Supplementary Materials - Index**

| **Supplementary Methods** |  |
| --- | --- |
| Abbreviations | *page 4* |
| Table S1. Results from previous studies comparing outcomes of inflammatory bowel disease after bariatric surgery. | *page 5* |
| Table S2. Overview of data sources | *page 7* |
| Table S3. ICD codes for exclusion criteria | *page 8* |
| Table S4. *International Classification of Disease* (ICD) codes and SNOMED codes defining inflammatory bowel diseases (IBD). | *page 9* |
| Table S5. Montreal classification of location of Crohn’s disease and extent of ulcerative colitis | *page 10* |
| Table S6. ICD codes for primary sclerosing cholangitis | *page 11* |
| Table S7. Procedure codes for IBD surgery | *page 12* |
| Table S8. Drug exposure definitions in the Swedish National Patient Register, the Swedish Prescribed Drug Register, and the Swedish inflammatory bowel disease register (SWIBREG). | *page 13* |
| Table S9. ICD - and ATC codes used to define comorbidity. | *page 14* |
| Table S10. Disease severity variables used in propensity score matching | *page 15* |
| Table S11. Variabels included from Scandinavian Obesity Surgery Registry, SOREG. | *page 16* |
| **Supplementary Results** |  |
| Table S12. Baseline characteristics of patients before and after matching | *page 17* |
| Table S13. Postoperative complications within 30 days after surgery for patients with inflammatory bowel disease undergoing bariatric surgery | *page 20* |
| Figure S14. Kaplan-Meier failure curve of time to first IBD-related hospitalization, first systemic corticosteroid use, and start of immunomodulator | *page 21* |
| Figure S15. Kaplan-Meier failure curve of time to start of targeted therapy, and first major IBD-related surgery | *page 22* |
| Table S16. Baseline characteristics of patients before and after matching using Body Mass Index as a direct matching variable | *page 23* |
| Figure S17. Kaplan-Meier curves of time to composite outcome in patients with bariatric surgery vs matched controls after matching, including BMI as a direct matching variable | *page 26* |
| Table S18. Risk of IBD-related hospitalization, first systemic corticosteroid use, and first major IBD-related surgery in patients with bariatric surgery vs matched controls including BMI as a direct matching variable. | *page 27* |
| **References** | *page 28* |

**Supplementary Methods**

Abbreviations

| CI | Confidence interval |
| --- | --- |
| CD | Crohn’s disease |
| SG | Sleeve gastrectomy |
| GB | Gastric Banding |
| RYGB | Roux-en-Y gastric bypass |
| NA | Not available |
| IRR | Incidence rate ratio |
| IQR | Interquartile range |
| CI | Confidence intervall |
| aOR | Adjusted odds ratio |
| E | Extent |
| HR | Hazard ratio |
| IBD | Inflammatory bowel disease |
| IBD-U | IBD unclassified |
| ICD | International Classification of Diseases |
| SE | Sweden |
| TPR | Total Population Register |
| UC | Ulcerative colitis |
| L | Location |
| PSC | Primary Sclerosing Cholangitis |

**Table S1.** Results from previous studies comparing outcomes of inflammatory bowel disease after bariatric surgery.

| **Study** | **Country/ Study type** | **Study period** | **N and subtype of patients followed** | **Intervention group** | **Control group** | **Follow-up time** | **IBD outcomes** |
| --- | --- | --- | --- | --- | --- | --- | --- |
| **Current study** |  |  |  |  |  |  |  |
| Stenberg et al, 2024 | Sweden | 2007-2020 | CD=145  UC=238 | SG=208  RYGB=191 | N=399 | Median follow-up:  I: 3.3 yrs  C:3.0 yrs | Composite endpoint:  HR 0.66 (95%CI 0.51-0.85) |
| Previous studies |  |  |  |  |  |  |  |
| Keidar 2015 ^1^  Surg Obes Rel Dis | USA, Retrospective cohort | 2006-2014 | CD=8  UC=2 | SG=9  GB=1 | - | Mean follow-up:  3.1 yrs (range 0.2-5-5) | IBD exacerbation= 3 (30%)  De-escalation of medication=3 (30%) |
| Aminian 2016 ^2^  Obes Surg | USA, Retrospective cohort | 2005-2012 | CD=7  UC=13 | SG=9  RYGB=7  GB=3  Revision=1 | - | Median follow-up:  2.9 ± 1.8 yrs | Reduction of symtoms or medication=9 (45%)  Acute flare of IBD=2 (10%) |
| Sharma, 2018 ^3^  Obes Surg | USA,  Unmatched cohort | 2004-2014 | 35-43 | N=493 | N=14826 | NA | UC , IRR (95%CI)  Strictures: 2.3 (1.4-3.7)  Fistulae: 0.6 (0.1-4.4)  Malnutrition: 0.2 (0.03-1.5)  Renal failure: 0.6 (0.01-0.42)  CD, IRR (95%CI)  Strictures: 1.1 (0.7-1.6)  Fistulae: NA  Malnutrition: 0.2 (0.03-1.4)  Renal failure: 0.1 (0.01-0.5) |
| Aelfers, 2018 ^4^  Obes Surg | Netherlands, Retrospective cohort | 1995-2016 | CD=29  UC=16 | SG=23  RYGB=9  GB=6  Revision=7 | - | Median follow-up:  3.9 ± 3.0 yrs | Exacerbation of IBD=3 (6.7%) |
| Hudson, 2019 ^5^  Inflamm Intest Dis. | USA,  Retrospective cohort | 2006-2018 | CD=9  UC=4 | SG=9  RYGB=3  GB=1 | - | NA | Deescalation of immunosuppression=2 (15%)  Early flare in IBD=0 (0%) |
| Heshmati, 2019 ^6^  J Am Coll Surg | USA,  Retrospective cohort | 2000-2017 | CD=31  UC=23 | SG=35  RYGB=19 | - | 1-yr follow-up | Improvement in medication CD:  SG=44%; RYGB=37.5%  Improvement in medication UC:  SG=8%; RYGB=27%  Worsened medication CD:  SG=3%; RYGB=26%  Worsened medication UC:  SG=0%; RYGB=9% |
| McKenna, 2020 ^7^  Obes Surg | USA,  Retrospective cohort | 2006-2017 | CD=10  UC=20  Unspecified= 1 | SG=14  RYGB=14  GB=4 | - | Median follow-up:  2.7 yrs (IQR 0.8-4.2) | Increased immunosuppression=1/9  Decreased immunosuppression=2/9 |
| Braga Neto, 2020 ^8^  Inflamm Bowel Dis | USA,  Matched cohort | 1996-2016 | CD=12  UC=13 | N=25  RYGB=15  SG=2  GB=6  Other=2 | N=25 | Median  I=7.69 yrs  C=7.89 yrs | IBD-related outcomes  I=48%  C=72%  OR 0.44 (95%CI 0.1-1.60) |
| Reenaers, 2021 ^9^  Inflamm Bowel Dis | France,  Case-control | 2008-2020 | CD=66  UC=22 | SG=73  GB=12  RYGB=3 | - | Mean follow-up: 2.8 yrs | Treatment de-escalation= 3 (4%)  Treatment escalation (17%) |
| Corbière, 2023 ^10^  Br J Surg | France,  Retrospective cohort | 2016-2018 | CD=326  UC=261 | SG=476  RYGB=86  GB=20  Other=5 | - | 2-yrs follow-up | Unplanned readmission for IBD within 2 yrs=86 (14.7%) |
| Desai, 2024 ^11^  J Clin Gastroenterol. | USA,  Matched cohort study | 2004-2022 | CD=246  UC=124  CD=236  UC=98 | N=473  SG=68%  RYGB=32% | N=473 | 2-yrs follow-up | IV steroid use or IBD-related surgery (aOR)=0.56 (95%CI 0.41-0.76)  Biological or small molecule therapy  (aOR)=0.27 (95%CI 0.13-0.55) |

Abbreviations: IBD= Inflammatory Bowel Disease; CD=Crohn’s disease; UC=Ulcerative colitis; CI=confidence interval; aOR=adjusted Odds ratio; IRR=Incidence rate ratio; IQR= Interquartile range; SG= Sleeve gastrectomy; RYGB= Roux-en-Y gastric bypass; GB=Gastric banding;
**• The summary of studies was based on a structured review of the literature including randomized control-trials, cohort studies, case-control studies and case-reports of at least 10 patients. We searched the PudMed in September 2023 using the following search terms “ (inflammatory bowel disease OR Crohn OR ulcerative colitis) AND (bariatric surgery OR gastric bypass OR sleeve gastrectomy). The search resulted in 242 studies. After review of titel and abstract, 21 studies remained. After review of full text articles the 11 articles presented in the table remained.**

**Table S2.** Overview of data sources

| **Register name** | **Administrator** | **Type of data** | **Coverage^[[1]](#footnote-1)^** |
| --- | --- | --- | --- |
| Total Population Register ^12^ ^13^ | Statistics Sweden | Data on all persons registered in Sweden, including birthdate, sex, place of residence, dates of last immigration and emigration dates. | 1968-2022 |
| Swedish National Patient Register ^14^ | Swedish National Board of Health and Welfare | Data from outpatient and inpatient contacts in hospital-based care, including date of contact, primary and secondary diagnoses and procedures | 1964-2022 ^b^ |
| The Swedish Prescribed Drug Register ^15^ | Swedish National Board of Health and Welfare | All dispensed prescriptions in Sweden | 1 July 2005-2022 |
| ESPRESSO Pathology Database ^16^ | Karolinska Institutet | Histopathology data retrieved from all of Sweden’s 28 pathology registers. | 1965-2017 |
| Swedish Cancer Register^17^ | Swedish National Board of Health and Welfare | Lymphoma | 1958-2021 |
| Swedish Cause of Death Register ^18^ | Swedish National Board of Health and Welfare | Main and underlying causes of death | 1952-2022 |
| Swedish IBD quality register (SWIBREG) ^19^ | Region Jönköping | Exposure to infusion drugs, BMI, and smoking data | 2005-2022 |
| Scandinavian Obesity Surgery Registry (SOREG) ^20^ | Region Örebro County | Baseline data, perioperative data and follow-up data for virtually all patients operated with bariatric surgery in Sweden. | 2007-2022 |

Time period used for the current study, not necessarily reflecting data availability.

^b^ outpatient surgery since 1997 and outpatient visits since 2001

Table S3. ICD codes for exclusion criteria

|  | **ICD-10** | **Procedure code** |
| --- | --- | --- |
| Chronic hepatitis | B18, K73 |  |
| Chronic liver disease | K72, R18, I85 |  |
| Transplantation | Z94 | DR008, DR010 |
| Chronic kidney disease (glomerulonephritis, dialysis, kidney transplantation) | N18.3-N18.5, N03, N05, Z49.1, Z49.2, Z99.2 | KAS00, KAS10, KAS20 |
| Liver cirrhosis | K70.3, K71.7, K74.3–K74.6 |  |
| Alcohol related diseases other than liver cirrhosis | F10, I42.6, G62.1, K29.2, G72.1, G31.2, K86.0, K85.2 |  |
| *ACS<6 months* |  |  |
| Unstable angina | I20.0 |  |
| Non-ST-Elevation Myocardial Infarction (NSTEMI) | I21.4; I21.4A; I21.4B; I21.4W; I21.4X |  |
| ST-elevation myocardial infarction (STEMI) | I21.0; I21.1; I21.2; I21.3; |  |
| Unspecific myocardial infarction | I21.9 |  |
| Colectomy in ulcerative colitis |  | (Table S6) |
| Presence of ileostoma |  | (Table S6) |
| Diagnosis of upper GI cancer | C15, C16, C22-C25 |  |
| Any cancer diagnosis <1y (except NMSC) | C00-C97 |  |

NMSC, non-melanoma skin cancer

Table S4. *International Classification of Disease* (ICD) codes and SNOMED codes defining inflammatory bowel diseases (IBD).

|  | **ICD-7** | **ICD-8** | **ICD-9** | **ICD-10** | **SNOMED codes*** |
| --- | --- | --- | --- | --- | --- |
| **Swedish National Patient Register** | **1964-1968** | **1969-1986** | **1987-1996** | **1997-** | **1965-** |
| Ulcerative colitis (UC) | 572,20;  572,21;  578,03 | 569,04  563,1; 563,10; 569,02 | 556 | K51 | D6255 *or* M41, M42, M43, M44, M463, or M47 |
| Crohn’s disease (CD) | 572,00;  572,09 | 563,00 | 555 | K50 | D6216 *or* M41, M42, M43, M44, M463, or M47 |
| IBD unclassified (IBD-U) | UC+CD | UC+CD  Or either of:  563; 563,0; 563,9; 563,98; 563,99 | UC+CD | UC + CD,  or K52.3 | D6214 *or* M41, M42, M43, M44, M463, or M47 |

ICD codes are captured prospectively in routine medical practice in the Swedish National Patient Registers, as described in several previous reports^21 22^. In Sweden, universal access to publicly funded health care is available to all residents, independent of residence, socioeconomic status, and disease severity. Having ≥1 *International Classification of Disease* codes for IBD plus a relevant biopsy code has a positive predictive value of 95-97%^23-26^.

*SNOMED codes starting with “M” (inflammation suggestive of IBD, but not a specified subtype) were required to be accompanied by a topographic code of T67 or T68 (colon). M41 refers to all codes starting with “M41” etc. D codes are diagnostic codes but listed under morphology in pathology registers. D6216 is the diagnostic code for “Crohn’s disease”.

Because definitions of exposure should not “look into the future”, IBD subtypes were defined by the two last diagnostic lisitings before bariatric surgery or match date. For individuals with one ICD code for IBD and one SNOMED code, the IBD subtype was determined by the ICD code only. In a recent paper (Everhov *et al.*, 2019 ^27^), we report that 18% of incident IBD patients in the Swedish patient register during 2002-2014 were classified as another IBD subtype at some point during follow-up. In a validation study of some 1,400 confirmed IBD patients on biologics (Shrestha et al., 2020), we found the positive predictive value for IBD subtypes defined by all available register information at end of follow-up to be 97% (95%CI 96-99) for CD and 98% (97-100) for UC. In the same study, we found the age groups (i.e., A1, A2, and A3 according to the Montreal classification), based on first IBD diagnostic listing in the National Patient Register to have a positive predictive value of 95% (93–98) for A1, 96% (95–97) for A2, and 90% (86–94) for A3.

Duration between first and second record indicative of IBD have been reported in detail elsewhere (Olén, et al, Lancet 2020 and Olén et al, Lancet Gastroenterology and Hepatology 2020). The median time between first and second “hit” was longer in earlier calendar periods than in more recent ones and in Sweden varied between 1.2-1.7 years in the 70-80-ies, 0.1-0.7 years in the 90-ies, and 0.04-0.09 years (15-33 days) since 2003.

**Table S5.** Montreal classification of location of Crohn’s disease and extent of ulcerative colitis.^28^

Definitions and diagnostic codes used to define IBD phenotypes according to the Montreal classification since the start of ICD-10.

| **Crohn’s disease** | **Location/Behavior** | **ICD-10** |
| --- | --- | --- |
| L1 | Small bowel disease; Terminal ileitis (excluding colon engagement) | K50.0 |
| L2 | Colon (excluding small bowel engagement) | K50.1 |
| L3/LX | Inflammation in both small and large bowel; Ileocecal Crohn’s disease; Location not defined | K50.8, K50.9 |
| B1 | Non-stricturing, non-penetrating | None of the ICD-codes for B2 or B3. |
| B2 | Stricturing | Crohn’s disease AND any of the following codes (K56.5; K56.6; K56.7; K62.4) |
| B3 | Penetrating | Crohn’s disease AND any of the following diagnostic codes (K63.0, K63.2, K31.6, N82.3, N82.3, N82.4) OR any of the following surgical procedure codes (JFA76, JFA86). |
| P^*^ | Perianal disease modifier | Crohn’s disease AND any of the following diagnostic codes: (K60.3, K60.4, K60.5, K61.0, K61.1, K61.2, K61.3, K61.4, K62.4) OR any of the following surgical procedure codes: (JHD20, JHD30, JHD33, JHD50, JHD60, JHD63, JHA00, JHA20, JHW96) |
| **Ulcerative colitis** | **Extent** |  |
| E1 | Ulcerative proctitis | K51.2 |
| E2 | Left-sided colitis | K51.3; K51.5 |
| E3 | Extensive colitis | K51.0 |
| Ex | Extent not specified | K51.4; K51.8; K51.9 |

^*^A recent validation study of use of ICD-codes in National Patient Register showed a positive predictive value (PPV) of 97 (96-99) % for the diagnosis of Crohn’s disease but lower for phenotypes of the Montreal classifications scheme. We have not classified included patients into phenotype except for Crohn’s disease with perianal manifestations, where the PPV was 83 (78-87)% ^29^

L=Location; E=extent; UC=ulcerative colitis

Validated* definitions and diagnostic codes used to define Crohn’s disease according to the Montreal classification since the start of use of the *International Classification of Diseases, Tenth revision* (ICD-10) (1994 in Denmark and 1997 in Sweden). All codes are captured in the Danish and Swedish National Patient Registers (prospectively recorded in routine clinical practice).
* Shrestha S, Olén O, et al. Scand J Gastroenterol. 2020 Apr;55(4):430-435. PMID: 32370571.
In that study, we found the positive predictive values to be the following: E1/E2: 80% (71-89), E3: 82% (78-87), non-L2: 81% (76-85), and L2: 36% (32-40).

Table S6. ICD codes for primary sclerosing cholangitis

|  | **ICD-9** | **ICD-10** |
| --- | --- | --- |
| **Sweden** | **1987-1996** | **1997-** |
| Primary sclerosing cholangitis^a^ | 576B | K830A |

*International Classification of Diseases, Ninth and Tenth Revisions* (ICD-9 and ICD-10) codes as recorded prospectively in the Danish and Swedish National Patient Registers. We restricted our use to ICD-9 and ICD-10 codes since we believe that earlier ICD codes for extraintestinal inflammation are less reliable, particularly for primary sclerosing cholangitis.

^a^ The validity of the PSC codes has not formally been tested in Sweden but according to Professor Annika Bergquist ([annika.bergquist@ki.se](mailto:annika.bergquist@ki.se), a renowned researcher in PSC epidemiology at Karolinska Institutet and also the PI for a large Swedish PSC-cohort and biobank) the impression is that the sensitivity and positive predictive value of K830A (=”sclerosing cholangitis”) for identifying PSC in IBD in the National Patient Register is not perfect but the nationwide prevalence of PSC in IBD in the Patient Register is roughly as would be expected, based on the prevalence in detailed patient cohorts at the university hospitals. However, since the PSC variable has not been validated and the corresponding ICD-code is not PSC-specific, some degree of misclassification can be expected.

**Table S7**. Procedure codes for IBD surgery

|  | **6^th^ version of Swedish surgery codes** | **NOMESCO** |
| --- | --- | --- |
| **Sweden** | **1985-1996** | **1997-** |
| **Major surgery** |  |  |
| Total colectomies including proctocolectomies | 465x | JFHxx |
| Other intestinal resections (small and large intestine and rectal) | 463x, 464x, 4810, 482x | JFBxx |
| Ileostoma |  | JFF10, JFF11, JFF13,  JFH33 and NOT  JFG00, JFG10, JFG20, JFG23, JFG26, JFG29, JFG30, JFG33, JFG36,  JFC40, JFC41 |
| Colostoma or unknown location |  | JGB10, JGB11, JFF23, JFF23, JFF26, JFF27, JFF30, JFF31, JFB60, JFB61, JFB63, JFB64, JFF96, JFF97 and NOT  JFG00, JFG10, JFG20, JFG23, JFG26, JFG29, JFG30, JFG33, JFG36,  JFC40, JFC41 |
| **Minor surgery** |  |  |
| Perianal surgery |  | JHD20, JHD30, JHD33, JHD50, JHD60, JHD63, JHA00, JHA20, JHW96 |

**x indicates any number or letter.**

In a validation study of surgery/procedure codes in the Swedish National Patient Register, we reviewed medical records of 155 IBD patients undergoing surgery and found the positive predictive value of surgery codes to be high (98.7%; 95%CI=95.4-99.8)^30^

**Table S8.** Drug exposure definitions in the Swedish National Patient Register, the Swedish Prescribed Drug Register, and the Swedish inflammatory bowel disease register (SWIBREG).

| **Drug group** | **Substance (indication)** | **ATC-code** | **Conversion factor to prednisolone equivalents** |
| --- | --- | --- | --- |
| **Biologics** |  |  |  |
| **Anti-TNFα** | Infliximab (UC/CD) | L04AB02 (L04AA12 before 2008) |  |
|  | Adalimumab (UC/CD) | L04AB04 (L04AA17 before 2008) |  |
|  | Golimumab (UC) | L04AB06 |  |
| **α4β7 integrin-inhibitor** | Vedolizumab (UC/CD) | L04AA33 |  |
| **Inhibitors of interleukin 12 and 23** | Ustekinumab (UC/CD) | L04AC05 |  |
| **Immunomodulators** |  |  |  |
|  | Azathioprine (UC/CD) | L04AX01 |  |
|  | Mercaptopurine (UC/CD) | L01BB02 |  |
|  | Methotrexate (CD) | L04AX03/L01BA01 |  |
| **Systemic corticosteroids** |  |  |  |
|  | Betamethasone | H02AB01 | 8.33 |
|  | Dexamethasone | H02AB02 | 6.67 |
|  | Methylprednisolone | H02AB04 | 1.25 |
|  | Prednisolone | H02AB06 | 1 |
|  | Prednisone | H02AB07 | 1 |

ATC: anatomical therapeutic chemical classification; CD: Crohn’s disease; UC: ulcerative colitis

**Table S9**. ICD - and ATC codes used to define comorbidity.

| **Comorbidity** | **ICD-code** | **ATC-code** |
| --- | --- | --- |
| Stable angina or previous acute cardiac event | I20-22 | B01AC06, B01AC04, B01AC24, B01AC22, C01DA |
| Chronic obstructive pulmonary disease | J41-J44 |  |
| Cerebrovascular disease | I60-I69 |  |
| Venous thromboembolic disease | I26, I80-I82 | B01AA03, B01AE07, B01AF |
| Peripheral arterial disease | I73 | B01AC06, B01AC04, B01AC24, B01AC22, C10AA |
| Arrhythmias | I441; I442; I452; I453; I456; I459; I460; I461; I469; I470; I472; I48; I471; I490; I495; R960 |  |
| Congestive heart failure | I42; I50 |  |
| Diabetes mellitus | E10-14, O24 | A10 |
| Hypertension | I10-I15 | C03, C07, C08, C09 |

For stable angina, venous thromboembolic disease, peripheral arterial disease, and arrhythmia we required ≥1 ICD and ≥1 ATC code. For cerebrovascular disease, we required either a hospitalization or a minimum 2 visits at a neurology or internal medicine clinic. A diagnosis of congestive heart failure and chronic obstructive pulmonary disease required minimum 2 diagnoses (in- or outpatient visits). For diseases diabetes mellitus and hypertension, we used either ≥2 diagnoses in the National Patient Register or ≥2 dispensings in the Prescribed Drug Register for a related medication

ICD = International Statistical Classification of Disease and Related Health Problems; ATC = anatomical therapeutic chemical classification

**Table S10**: Disease severity variables used in propensity score matching

|  | Crohn’s disease | Ulcerative colitis |
| --- | --- | --- |
| *Line of targeted therapy* |  |  |
| 0 | 0 | 0 |
| 1 | 1 | 1 |
| ≥2 | 2 | 2 |
| Immunomodulator therapy (ever) | 1 | 1 |
| Cumulative oral corticosteroid dose within 2 years |  |  |
| 0 | 0 | 0 |
| 0<-1500 mg | 1 | 1 |
| 1500<-3000 mg | 2 | 2 |
| 3000<-4500 mg | 3 | 3 |
| 4500 mg | 4 | 4 |
| Previous intestinal surgery (except appendectomy) | 3 | - |

Table S11. Variabels included from Scandinavian Obesity Surgery Registry, SOREG.

| **Variable** | **SOReg variable** (in Swedish language) |
| --- | --- |
| BMI before surgery | BMI |
| Age at the time of surgery | Ålder vid operation |
| Type of surgery | Operationsmetod |
| Index data (operation date) | Operationsdatum |
| Surgical access | Kirurgisk access |
| Intraoperative complication | Peroperativa komplikationer |
| Type of intraoperative complication | Mjältskada; Oavsiktlig tarmperforation; Vilken annan peroperativ komplikation |
| Postoperative complication within 30 days of surgery | Postoperativ komplikation |
| Severity of complication | Komplikationens/ernas svårighetsgrad |
| Totalt weight loss (TWL) at 1 year after surgery | TWL(%) (1 år) |
| Total weight loss (TWL) at 2 years after surgery | TWL(%) (2 år) |
| Body Mass Index (BMI) loss at 1 year after surgery | BMI minskning (1år) |
| BMI loss at 2 years after surgery | BMI minskning (2år) |

**Supplementary Results**

**Table S12.** Baseline characteristics of patients before and after matching.

| **Characteristic** | **Before matching*** | | | **After matching**** | | |
| --- | --- | --- | --- | --- | --- | --- |
|  | **Surgery** | **No bariatric surgery** | **Standardized mean difference** | **Surgery** | **Matched controls** | **Standardized mean difference** |
| N | 455 | 2 811 |  | 399 | 399 |  |
| Sex, n (%) |  |  |  |  |  |  |
| Women | 367 (80.7) | 1404 (49.9) | 0.682 | 316 (79.2) | 314 (78.7) | 0.012 |
| Men | 88 (19.3) | 1407 (50.1) | 0.682 | 83 (20.8) | 85 (21.3) | 0.012 |
| Age at IBD diagnosis (years) |  |  |  |  |  |  |
| Mean (SD) | 33.1 (10.7) | 37.3 (14.8) | 0.327 | 33.5 (10.7) | 33.0 (10.7) | 0.043 |
| Median (IQR) | 33.2 (25.3-40.9) | 35.8 (25.5-48.6) |  | 33.7 (25.7-40.9) | 32.6 (25.4-39.8) |  |
| Range, min-max | 7.8-61.2 | 1.0-73.8 |  | 7.8-61.2 | 7.8-63.2 |  |
| *Categories, n (%)* |  |  |  |  |  |  |
| <18y | 40 (8.8) | 248 (8.8) | 0.001 | 32 (8.0) | 33 (8.3) | 0.009 |
| 18-<40y | 291 (64.0) | 1423 (50.6) | 0.272 | 255 (63.9) | 269 (67.4) | 0.074 |
| 40-<60y | 123 (27.0) | 913 (32.5) | 0.119 | 111 (27.8) | 94 (23.6) | 0.098 |
| ≥60y | 1 (0.2) | 227 (8.1) | 0.402 | 1 (0.3) | 3 (0.8) | 0.071 |
| Year at IBD diagnosis, n (%) |  |  |  |  |  |  |
| 1969-1990 | 33 (7.3) | 243 (8.6) | 0.051 | 26 (6.5) | 30 (7.5) | 0.039 |
| 1991-2000 | 78 (17.1) | 425 (15.1) | 0.055 | 70 (17.5) | 75 (18.8) | 0.033 |
| 2001-2010 | 235 (51.6) | 1264 (45.0) | 0.134 | 203 (50.9) | 212 (53.1) | 0.04.5 |
| 2011-2020 | 109 (24.0) | 879 (31.3) | 0.164 | 100 (25.1) | 82 (20.6) | 0.108 |
| Age at index date (years) |  |  |  |  |  |  |
| Mean (SD) | 43.3 (9.9) | 46.8 (14.6) | 0.279 | 43.8 (10.0) | 43.9 (10.1) | 0.009 |
| Median (IQR) | 43.5 (36.1-50.5) | 47.2 (35.2-58.7) |  | 44.0 (36.7-51.1) | 44.4 (36.6-51.1) |  |
| Range, min-max | 20.6-69.9 | 18.0-74.8 |  | 20.6-69.9 | 18.3-69.7 |  |
| *Categories, n (%)* |  |  |  |  |  |  |
| 18-<40y | 180 (39.6) | 966 (34.4) | 0.108 | 152 (38.1) | 146 (36.6) | 0.031 |
| 40-<60y | 255 (56.0) | 1208 (43.0) | 0.264 | 228 (57.1) | 231 (57.9) | 0.015 |
| 60y-<75y | 20 (4.4) | 637 (22.7) | 0.554 | 19 (4.8) | 22 (5.5) | 0.034 |
| Year at index date, n (%) |  |  |  |  |  |  |
| 2007-2012 | 157 (34.5) | 764 (27.2) | 0.159 | 123 (30.8) | 123 (30.8) | 0.0 |
| 2013-2016 | 159 (34.9) | 1102 (39.2) | 0.088 | 142 (35.6) | 142 (35.6) | 0.0 |
| 2017-2020 | 139 (30.5) | 945 (33.6) | 0.066 | 134 (33.6) | 134 (33.6) | 0.0 |
| Disease duration at index date |  |  |  |  |  |  |
| Mean (SD) | 10.3 (8.2) | 9.5 (8.8) | 0.086 | 10.3 (8.1) | 10.9 (7.4) | 0.070 |
| Median (IQR) | 8.5 (4.2-14.1) | 7.5 (2.4-13.9) |  | 8.6 (4.2-14.5) | 9.9 (4.8-14.9) |  |
| Range, min-max | 0.0-45.7 | 0.0-46.9 |  | 0.0-45.7 | 0.1-44.1 |  |
| *Categories, n (%)* |  |  |  |  |  |  |
| 0-<1y | 31 (6.8) | 421 (15.0) | 0.264 | 27 (6.8) | 15 (3.8) | 0.135 |
| 1-<5y | 104 (22.9) | 661 (23.5) | 0.016 | 88 (22.1) | 87 (21.8) | 0.006 |
| 5-<10y | 131 (28.8) | 610 (21.7) | 0.164 | 116 (29.1) | 102 (25.6) | 0.079 |
| ≥10y | 189 (41.5) | 1119 (39.8) | 0.035 | 168 (42.1) | 195 (48.9) | 0.136 |
| BMI at index date |  |  |  |  |  |  |
| Mean (SD) | 40.7 (5.2) | 33.7 (5.1) | 1.365 | 40.6 (5.3) | 34.0 (5.5) | 1.231 |
| Median (IQR) | 40.0 (37.1-43.8) | 32.0 (30.8-34.5) |  | 40.1 (37.0-43.9) | 32.1 (30.9-34.6) |  |
| Range, min-max | 30.1-63.6 | 30.0-67.9 |  | 30.1-63.6 | 30.0-62.5 |  |
| *Categories, n (%)* |  |  |  |  |  |  |
| 30-<35 | 51 (11.2) | 2190 (77.9) | 1.810 | 48 (12.0) | 304 (76.2) | 1.693 |
| 35-<40 | 172 (37.8) | 422 (15.0) | 0.535 | 147 (36.8) | 63 (15.8) | 0.492 |
| ≥40 | 232 (51.0) | 199 (7.1) | 1.105 | 204 (51.1) | 32 (8.0) | 1.072 |
| Education level (years), n (%) |  |  |  |  |  |  |
| <9 | 53 (11.6) | 544 (19.4) | 0.214 | 42 (10.5) | 47 (11.8) | 0.040 |
| 10-12 | 281 (61.8) | 1441 (51.3) | 0.213 | 251 (62.9) | 220 (55.1) | 0.158 |
| >12 | 121 (26.6) | 802 (28.5) | 0.043 | 106 (26.6) | 131 (32.8) | 0.137 |
| Missing | 0 (0.0) | 24 (0.9) | 0.131 | 0 (0.0) | 1 (0.3) | 0.071 |
| Country of birth |  |  |  |  |  |  |
| Nordic | 427 (93.8) | 2475 (88.0) | 0.203 | 372 (93.2) | 361 (90.5) | 0.101 |
| Non-Nordic | 26 (5.7) | 297 (10.6) | 0.178 | 25 (6.3) | 32 (8.0) | 0.068 |
| Missing | 2 (0.4) | 39 (1.4) | 0.100 | 2 (0.5) | 6 (1.5) | 0.101 |
| Region of residence (healthcare regions), n (%) |  |  |  |  |  |  |
| North | 37 (8.1) | 153 (5.4) | 0.107 | 33 (8.3) | 12 (3.0) | 0.230 |
| Central | 112 (24.6) | 317 (11.3) | 0.353 | 100 (25.1) | 35 (8.8) | 0.445 |
| Stockholm | 87 (19.1) | 707 (25.2) | 0.146 | 72 (18.0) | 71 (17.8) | 0.007 |
| West | 82 (18.0) | 424 (15.1) | 0.079 | 73 (18.3) | 83 (20.8) | 0.063 |
| South-east | 40 (8.8) | 673 (23.9) | 0.418 | 32 (8.0) | 97 (24.3) | 0.454 |
| South | 97 (21.3) | 536 (19.1) | 0.056 | 89 (22.3) | 101 (25.3) | 0.071 |
| Missing | 0 (0.0) | 1 (0.0) | 0.027 | 0 | 0 | - |
| IBD subtype, n (%) |  |  |  |  |  |  |
| CD | 153 (33.6) | 1225 (43.6) | 0.206 | 145 (36.3) | 145 (36.3) | 0.0 |
| UC | 260 (57.1) | 1435 (51.0) | 0.123 | 238 (59.6) | 238 (59.6) | 0.0 |
| IBD-U | 42 (9.2) | 151 (5.4) | 0.149 | 16 (4.0) | 16 (4.0) | 0.0 |
| Montreal stage in CD up until index date, n (%) |  |  |  |  |  |  |
| L2 | 23 (15.0) | 277 (22.6) | 0.195 | 22 (15.2) | 30 (20.7) | 0.144 |
| L1/L3/LX | 110 (71.9) | 805 (65.7) | 0.134 | 103 (71.0) | 97 (66.9) | 0.090 |
| Missing | 20 (13.1) | 143 (11.7) | 0.042 | 20 (13.8) | 18 (12.4) | 0.041 |
| Perianal | 17 (11.1) | 196 (16.0) | 0.143 | 15 (10.3) | 19 (13.1) | 0.086 |
| Montreal stage in UC up until index date, n (%) |  |  |  |  |  |  |
| E1/E2 (ulcerative proctitis/(left sided UC) | 100 (38.5) | 548 (38.2) | 0.006 | 89 (37.4) | 104 (43.7) | 0.129 |
| E3 (extensive UC) | 51 (19.6) | 565 (39.4) | 0.444 | 49 (20.6) | 79 (33.2) | 0.287 |
| EX (extent not defined) | 95 (36.5) | 282 (19.7) | 0.383 | 86 (36.1) | 48 (20.2) | 0.361 |
| Missing | 14 (5.4) | 40 (2.8) | 0.131 | 14 (5.9) | 7 (2.9) | 0.144 |
| Extraintestinal manifestations at start of follow-up, n (%) |  |  |  |  |  |  |
| Primary sclerosing cholangitis | 3 (0.7) | 66 (2.3) | 0.139 | 3 (0.8) | 11 (2.8) | 0.153 |
| Other extraintestinal manifestations | 89 (19.6) | 526 (18.7) | 0.022 | 81 (20.3) | 65 (16.3) | 0.104 |
| Comorbidity, n (%) |  |  |  |  |  |  |
| Ishemic heart disease | 6 (1.3) | 57 (2.0) | 0.055 | 6 (1.5) | 2 (0.5) | 0.101 |
| Congestive heart failure | 8 (1.8) | 24 (0.9) | 0.080 | 7 (1.8) | 2 (0.5) | 0.119 |
| Hypertension | 187 (41.1) | 870 (30.9) | 0.213 | 168 (42.1) | 94 (23.6) | 0.403 |
| Diabetes mellitus | 84 (18.5) | 269 (9.6) | 0.258 | 75 (18.8) | 31 (7.8) | 0.329 |
| Severity of IBD, n (%) |  |  |  |  |  |  |
| Previous types of targeted therapies |  |  |  |  |  |  |
| 0 | 410 (90.1) | 2051 (73.0) | 0.453 | 356 (89.2) | 364 (91.2) | 0.068 |
| 1 | 36 (7.9) | 567 (20.2) | 0.358 | 34 (8.5) | 27 (6.8) | 0.066 |
| ≥2 | 9 (2.0) | 193 (6.9) | 0.239 | 9 (2.3) | 8 (2.0) | 0.017 |
| Immunomodulator therapy ever before index date |  |  |  |  |  |  |
| Yes | 143 (31.4) | 1417 (50.4) | 0.393 | 139 (34.8) | 137 (34.3) | 0.011 |
| No | 312 (68.6) | 1394 (49.6) | 0.393 | 260 (65.2) | 262 (65.7) | 0.011 |
| Cumulative oral corticosteroids within 2 years before index date,  mg prednisolone equivalents |  |  |  |  |  |  |
| 0 | 338 (74.3) | 1651 (58.7) | 0.334 | 294 (73.7) | 298 (74.7) | 0.023 |
| 0<-1500 mg | 71 (15.6) | 545 (19.4) | 0.100 | 66 (16.5) | 64 (16.0) | 0.014 |
| 1500<-3000 mg | 27 (5.9) | 335 (11.9) | 0.211 | 24 (6.0) | 30 (7.5) | 0.060 |
| 3000<-4500 mg | 10 (2.2) | 142 (5.1) | 0.153 | 9 (2.3) | 4 (1.0) | 0.099 |
| ≥4500 mg | 9 (2.0) | 138 (4.9) | 0.161 | 6 (1.5) | 3 (0.8) | 0.071 |
| Previous intestinal surgery ever before index date |  |  |  |  |  |  |
| Yes | 32 (7.0) | 409 (14.5) | 0.244 | 27 (6.8) | 39 (9.8) | 0.109 |
| No | 423 (93.0) | 2402 (85.5) | 0.244 | 372 (93.2) | 360 (90.2) | 0.109 |
| Follow-up time primary outcome (years) |  |  |  |  |  |  |
| Mean (SD) | 4.6 (3.6) | 3.0 (3.2) | 0.464 | 4.2 (3.4) | 3.8 (3.2) | 0.146 |
| Median (IQR) | 3.8 (1.6-7.2) | 1.9 (0.4-4.8) |  | 3.3 (1.4-6.5) | 3.0 (1.1-5.7) |  |
| Range, min-max | 0.0-14.2 | 0.0-14.8 |  | 0.0-13.1 | 0.0-13.7 |  |
| *Categories, n (%)* |  |  |  |  |  |  |
| 0-<1y | 74 (16.3) | 1049 (37.3) | 0.489 | 70 (17.5) | 98 (24.6) | 0.173 |
| 1-<5y | 206 (45.3) | 1102 (39.2) | 0.123 | 195 (48.9) | 187 (46.9) | 0.040 |
| 5-<10y | 125 (27.5) | 540 (19.2) | 0.196 | 101 (25.3) | 90 (22.6) | 0.065 |
| ≥10y | 50 (11.0) | 120 (4.3) | 0.255 | 33 (8.3) | 24 (6.0) | 0.088 |
| Reason for censoring, n (%) |  |  |  |  |  |  |
| Primary outcome | 224 (49.2) | 1884 (67.0) | 0.367 | 201 (50.4) | 226 (56.6) | 0.126 |
| IBD-related hospitalization | 42 (9.2) | 482 (17.1) | 0.236 | 37 (9.3) | 46 (11.5) | 0.074 |
| Systemic corticosteroid use | 177 (38.9) | 1453 (51.7) | 0.259 | 158 (39.6) | 178 (44.6) | 0.102 |
| Start of immunomodulator | 89 (19.6) | 949 (33.8) | 0.325 | 82 (20.6) | 95 (23.8) | 0.078 |
| Addition or change of targeted therapy | 49 (10.8) | 751 (26.7) | 0.417 | 46 (11.5) | 59 (14.8) | 0.096 |
| Major IBD-related surgery | 19 (4.2) | 223 (7.9) | 0.158 | 14 (3.5) | 22 (5.5) | 0.097 |
| Death | 5 (1.1) | 19 (0.7) | 0.045 | 5 (1.3) | 0 (0.0) | 0.159 |
| Emigration | 3 (0.7) | 12 (0.4) | 0.032 | 3 (0.8) | 1 (0.3) | 0.071 |
| End of follow-up (December 31, 2021) | 223 (49.0) | 896 (31.9) | 0.355 | 190 (47.6) | 172 (43.1) | 0.091 |
| Surgical method, n (%) |  |  |  |  |  |  |
| Roux-en-Y gastric bypass | 235 (51.6) | - | - | 191 (47.9) | - | - |
| Sleeve gastrectomy | 220 (48.4) | - | - | 208 (52.1) | - | - |
| Propensity score |  |  |  |  |  |  |
| Mean (SD) | 0.24 (0.13) | 0.12 (0.11) | 0.999 | 0.51 (0.09) | 0.49 (0.07) | 0.146 |
| Median (IQR) | 0.24 (0.13-0.34) | 0.09 (0.04-0.17) |  | 0.50 (0.48-0.52) | 0.50 (0.48-0.51) |  |
| Range, min-max | 0.01-0.55 | 0.00-0.62 |  | 0.35-1.00 | 0.00-0.81 |  |

BMI: body mass index; CD: Crohn’s disease; IBD-U: Inflammatory bowel disease unclassified; IQR: Interquartile range; SD: standard deviation; UC: Ulcerative colitis

*Index date before matching: Date of bariatric surgery in the surgery group and first BMI measurement date after IBD diagnosis in the non-surgery group;

**Index date after matching: Date of bariatric surgery in the surgery group and the corresponding date in the matched controls

**Table S13.** Postoperative complications within 30 days after surgery for patients with inflammatory bowel disease undergoing bariatric surgery

| **Complication** | **Number (%)** |
| --- | --- |
| N | 399 |
| Postoperative complication | 32 (8.0%) |
| Leak or intraabdominal abscess | 9 (2.3%) |
| Bleeding | 6 (1.5%) |
| Abdominal wall complication^1^ | 2 (0.5%) |
| Bowel obstruction | 1 (0.3%) |
| Pulmonary complication | 2 (0.5%) |
| Cardiovascular complication | 0 (0.0%) |
| Marginal ulcer | 0 (0.0%) |
| Urinary tract infection | 1 (0.3%) |
| Other complication^2^ | 13 (3.3%) |

1. Including wound infection and wound dehiscence
2. Other complications, mainly abdominal pain, malnutrition or nausea/vomiting.


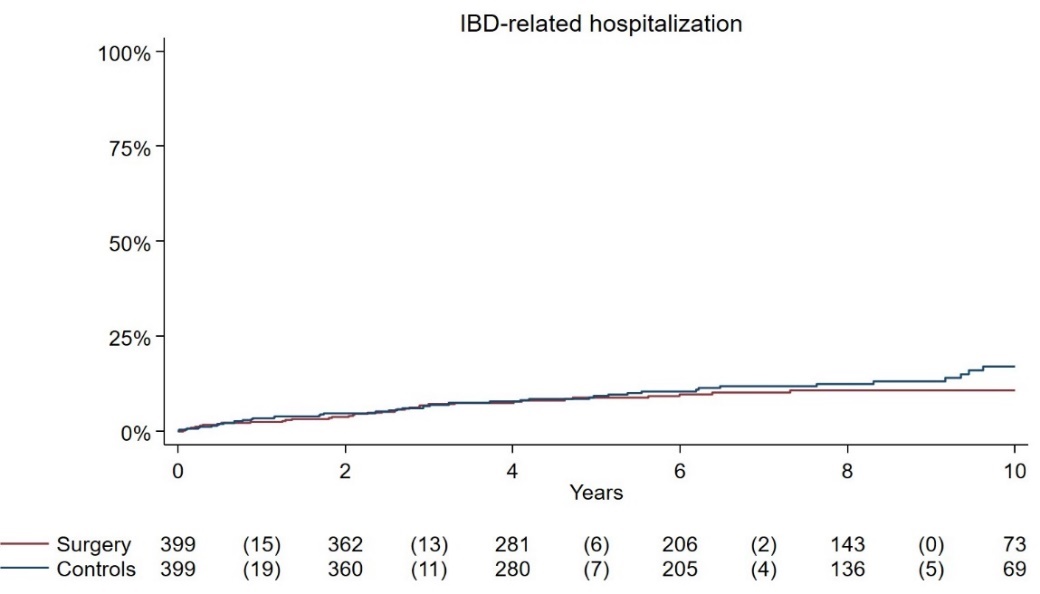


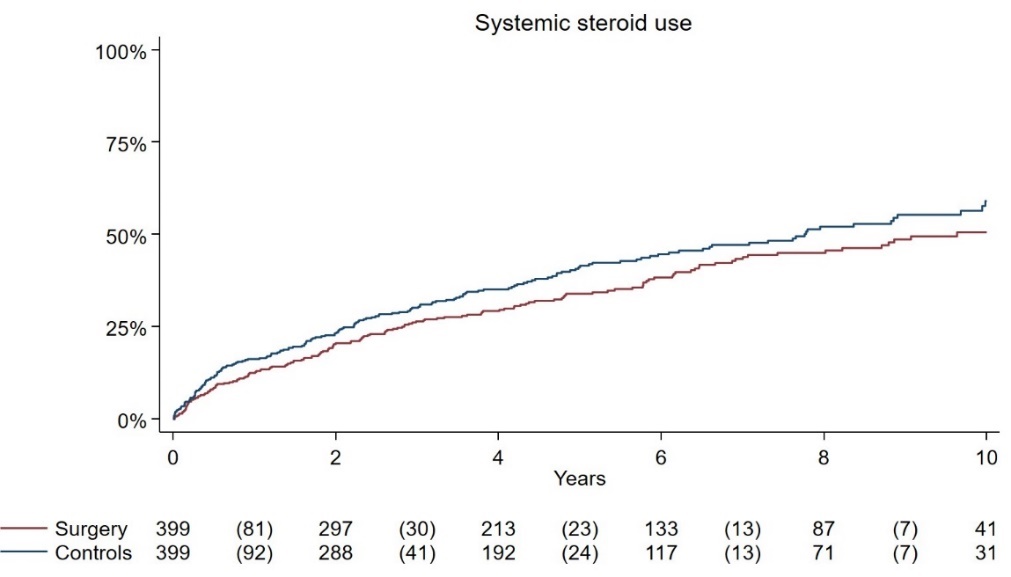


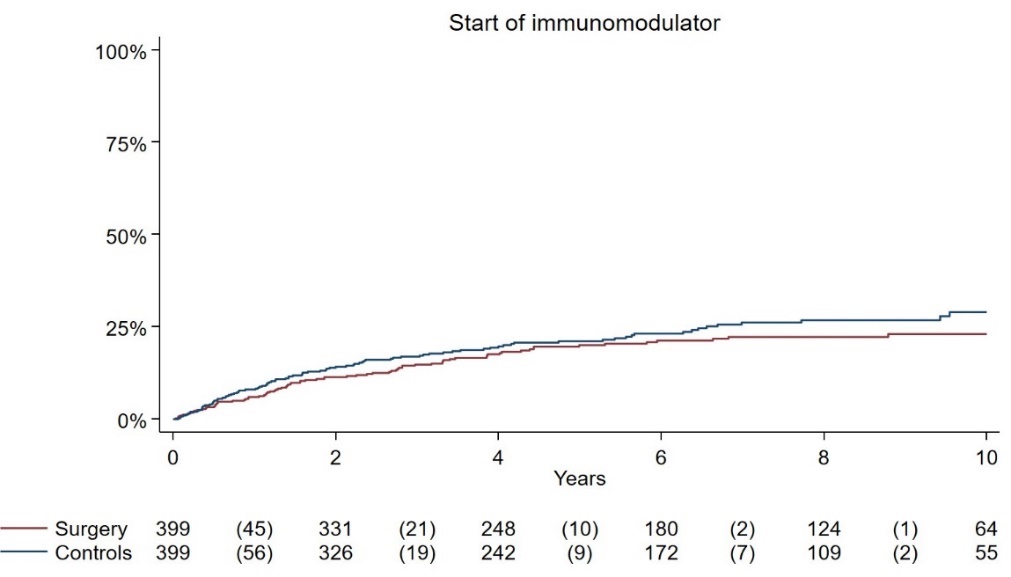


**Figure S14.** Kaplan-Meier failure curves of time to first IBD-related hospitalization (upper panel), first systemic corticosteroid use (middle panel), and start of immunomodulator (lower panel).


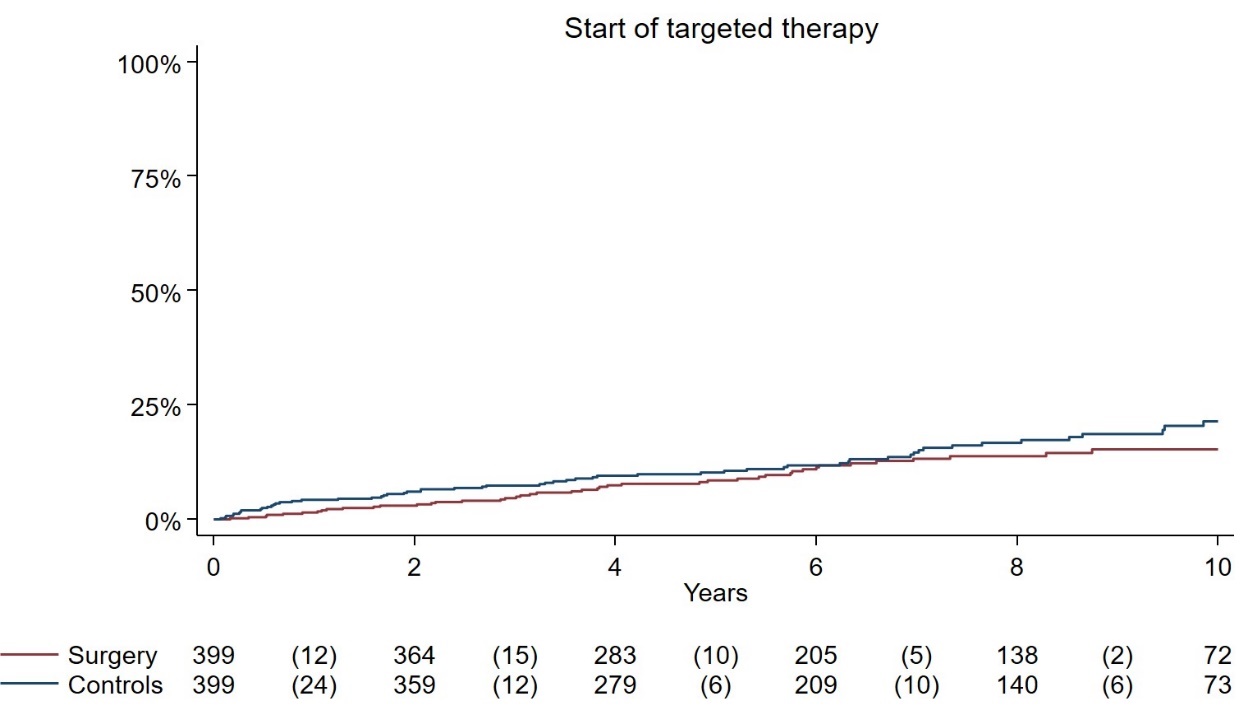


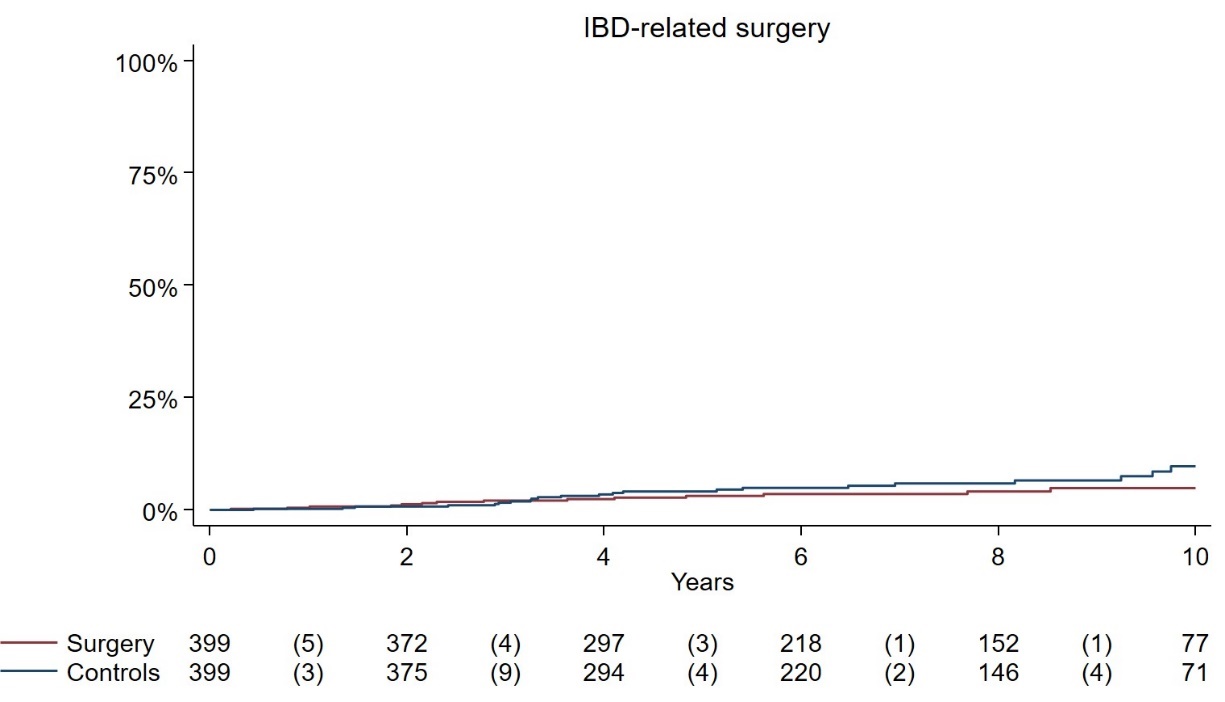


**Figure S15.** Kaplan-Meier failure curves of time to start of targeted therapy (upper panel) and first major IBD-related surgery (lower panel).

**Table S16.** Baseline characteristics of patients before and after matching using Body Mass Index as a direct matching variable

| **Characteristic** | **After matching*** | | |
| --- | --- | --- | --- |
|  | **Surgery** | **Matched controls** | **Standardized mean difference** |
| N | 221 | 221 |  |
| Sex, n (%) |  |  |  |
| Women | 175 (79.2) | 169 (76.5) | 0.065 |
| Men | 46 (20.8) | 52 (23.5) | 0.065 |
| Age at IBD diagnosis (years) |  |  |  |
| Mean (SD) | 33.9 (10.6) | 33.6 (11.3) | 0.028 |
| Median (IQR) | 34.1 (26.2-41.2) | 31.9 (25.6-41.8) |  |
| Range, min-max | 7.8-61.2 | 6.7-64.9 |  |
| *Categories, n (%)* |  |  |  |
| <18y | 18 (8.1) | 19 (8.6) | 0.016 |
| 18-<40y | 139 (62.9) | 141 (63.8) | 0.019 |
| 40-<60y | 63 (28.5) | 58 (26.2) | 0.051 |
| ≥60y | 1 (0.5) | 3 (1.4) | 0.096 |
| Year at IBD diagnosis, n (%) |  |  |  |
| 1969-1990 | 11 (5.0) | 15 (6.8) | 0.077 |
| 1991-2000 | 42 (19.0) | 40 (18.1) | 0.023 |
| 2001-2010 | 105 (47.5) | 107 (48.4) | 0.018 |
| 2011-2020 | 63 (28.5) | 59 (26.7) | 0.040 |
| Age at index date (years) |  |  |  |
| Mean (SD) | 44.8 (9.5) | 44.7 (9.9) | 0.008 |
| Median (IQR) | 45.8 (37.4-51.7) | 44.9 (38.4-50.8) |  |
| Range, min-max | 23.7-68.8 | 21.9-70.5 |  |
| *Categories, n (%)* |  |  |  |
| 18-<40y | 73 (33.0) | 69 (31.2) | 0.039 |
| 40-<60y | 137 (62.0) | 140 (63.3) | 0.028 |
| 60y-<75y | 11 (5.0) | 12 (5.4) | 0.020 |
| Year at index date, n (%) |  |  |  |
| 2007-2012 | 43 (19.5) | 43 (19.5) | 0.0 |
| 2013-2016 | 77 (34.8) | 77 (34.8) | 0.0 |
| 2017-2020 | 101 (45.7) | 101 (45.7) | 0.0 |
| Disease duration at index date |  |  |  |
| Mean (SD) | 10.9 (8.1) | 11.2 (7.9) | 0.028 |
| Median (IQR) | 9.2 (5.2-15.6) | 10.0 (5.3-15.2) |  |
| Range, min-max | 0.0-45.7 | 0.3-42.3 |  |
| *Categories, n (%)* |  |  |  |
| 0-<1y | 10 (4.5) | 8 (3.6) | 0.046 |
| 1-<5y | 43 (19.5) | 44 (19.9) | 0.011 |
| 5-<10y | 68 (30.8) | 60 (27.1) | 0.080 |
| ≥10y | 100 (45.2) | 109 (49.3) | 0.082 |
| BMI at index date |  |  |  |
| Mean (SD) | 38.8 (5.1) | 39.6 (7.8) | 0.113 |
| Median (IQR) | 38.1 (35.6-40.9) | 36.9 (35.2-41.4) |  |
| Range, min-max | 30.1-63.6 | 30.1-62.5 |  |
| *Categories, n (%)* |  |  |  |
| 30-<35 | 49 (22.2) | 49 (22.2) | 0.0 |
| 35-<40 | 98 (44.3) | 98 (44.3) | 0.0 |
| ≥40 | 74 (33.5) | 74 (33.5) | 0.0 |
| Education level (years), n (%) |  |  |  |
| <9 | 23 (10.4) | 27 (12.2) | 0.057 |
| 10-12 | 141 (63.8) | 121 (54.8) | 0.185 |
| >12 | 57 (25.8) | 72 (32.6) | 0.150 |
| Missing | 0 (0.0) | 1 (0.5) | 0.095 |
| Country of birth |  |  |  |
| Nordic | 202 (91.4) | 202 (91.4) | 0.0 |
| Non-Nordic | 18 (8.1) | 16 (7.2) | 0.034 |
| Missing | 1 (0.5) | 3 (1.4) | 0.096 |
| Region of residence (healthcare regions), n (%) |  |  |  |
| North | 20 (9.0) | 15 (6.8) | 0.084 |
| Central | 56 (25.3) | 24 (10.9) | 0.383 |
| Stockholm | 48 (21.7) | 43 (19.5) | 0.056 |
| West | 36 (16.3) | 31 (14.0) | 0.063 |
| South-east | 17 (7.7) | 58 (26.2) | 0.510 |
| South | 44 (19.9) | 50 (22.6) | 0.066 |
| Missing | 0 | 0 | - |
| IBD subtype, n (%) |  |  |  |
| CD | 80 (36.2) | 80 (36.2) | 0.0 |
| UC | 132 (59.7) | 132 (59.7) | 0.0 |
| IBD-U | 9 (4.1) | 9 (4.1) | 0.0 |
| Montreal stage in CD up until index date, n (%) |  |  |  |
| L2 | 13 (16.3) | 20 (25.0) | 0.218 |
| L1/L3/LX | 57 (71.3) | 50 (62.5) | 0.187 |
| Missing | 10 (12.5) | 10 (12.5) | 0.0 |
| Perianal | 7 (8.8) | 7 (8.8) | 0.0 |
| Montreal stage in UC up until index date, n (%) |  |  |  |
| E1/E2 (ulcerative proctitis/(left sided UC) | 53 (40.2) | 52 (39.4) | 0.015 |
| E3 (extensive UC) | 28 (21.2) | 48 (36.4) | 0.339 |
| EX (extent not defined) | 43 (32.6) | 27 (20.5) | 0.277 |
| Missing | 8 (6.1) | 5 (3.8) | 0.105 |
| Extraintestinal manifestations at start of follow-up, n (%) |  |  |  |
| Primary sclerosing cholangitis | 2 (0.9) | 10 (4.5) | 0.224 |
| Other extraintestinal manifestations | 52 (23.5) | 45 (20.4) | 0.077 |
| Comorbidity, n (%) |  |  |  |
| Ishemic heart disease | 4 (1.8) | 3 (1.4) | 0.036 |
| Congestive heart failure | 4 (1.8) | 2 (0.9) | 0.078 |
| Hypertension | 100 (45.2) | 71 (32.1) | 0.272 |
| Diabetes mellitus | 41 (18.6) | 24 (10.9) | 0.218 |
| Severity of IBD, n (%) |  |  |  |
| Previous types of targeted therapies |  |  |  |
| 0 | 191 (86.4) | 190 (86.0) | 0.013 |
| 1 | 23 (10.4) | 24 (10.9) | 0.015 |
| ≥2 | 7 (3.2) | 7 (3.2) | 0.0 |
| Immunomodulator therapy ever before index date |  |  |  |
| Yes | 83 (37.6) | 95 (43.0) | 0.111 |
| No | 138 (62.4) | 126 (57.0) | 0.111 |
| Cumulative oral corticosteroids within 2 years before index date,  mg prednisolone equivalents |  |  |  |
| 0 | 167 (75.6) | 164 (74.2) | 0.031 |
| 0<-1500 mg | 33 (14.9) | 29 (13.1) | 0.052 |
| 1500<-3000 mg | 11 (5.0) | 18 (8.1) | 0.128 |
| 3000<-4500 mg | 5 (2.3) | 3 (1.4) | 0.068 |
| ≥4500 mg | 5 (2.3) | 7 (3.2) | 0.056 |
| Previous intestinal surgery ever before index date |  |  |  |
| Yes | 17 (7.7) | 21 (9.5) | 0.065 |
| No | 204 (92.3) | 200 (90.5) | 0.065 |
| Follow-up time primary outcome (years) |  |  |  |
| Mean (SD) | 3.7 (3.0) | 3.2 (2.7) | 0.191 |
| Median (IQR) | 3.0 (1.4-5.3) | 2.6 (1.1-4.7) |  |
| Range, min-max | 0.0-13.0 | 0.0-12.3 |  |
| *Categories, n (%)* |  |  |  |
| 0-<1y | 37 (16.7) | 51 (23.1) | 0.159 |
| 1-<5y | 125 (56.6) | 123 (55.7) | 0.018 |
| 5-<10y | 49 (22.2) | 42 (19.0) | 0.078 |
| ≥10y | 10 (4.5) | 5 (2.3) | 0.125 |
| Reason for censoring, n (%) |  |  |  |
| Primary outcome | 106 (48.0) | 118 (53.4) | 0.109 |
| IBD-related hospitalization | 16 (7.2) | 20 (9.0) | 0.066 |
| Systemic corticosteroid use | 83 (37.6) | 89 (40.3) | 0.056 |
| Start of immunomodulator | 39 (17.6) | 48 (21.7) | 0.103 |
| Addition or change of targeted therapy | 21 (9.5) | 32 (14.5) | 0.154 |
| Major IBD-related surgery | 6 (2.7) | 9 (4.1) | 0.075 |
| Death | 2 (0.9) | 1 (0.5) | 0.055 |
| Emigration | 2 (0.9) | 1 (0.5) | 0.055 |
| End of follow-up (December 31, 2021) | 111 (50.2) | 101 (45.7) | 0.091 |
| Surgical method, n (%) |  |  |  |
| Roux-en-Y gastric bypass | 91 (41.2) | - | - |
| Sleeve gastrectomy | 130 (58.8) | - | - |
| Propensity score |  |  |  |
| Mean (SD) | 0.52 (0.08) | 0.48 (0.10) | 0.373 |
| Median (IQR) | 0.52 (0.47-0.55) | 0.52 (0.44-0.54) |  |
| Range, min-max | 0.34-0.76 | 0.00-0.77 |  |

BMI: body mass index; CD: Crohn’s disease; IBDU: IBD-U: Inflammatory bowel disease unclassified; IQR: Interquartile range; SD: standard deviation; UC: Ulcerative colitis

*Index date after matching: Date of bariatric surgery in the surgery group and the corresponding date in the matched control.

**Figure S17.** Kaplan-Meier failure curves of time to composite outcome in patients with bariatric surgery vs matched controls after matching including BMI as a direct matching variable.

**
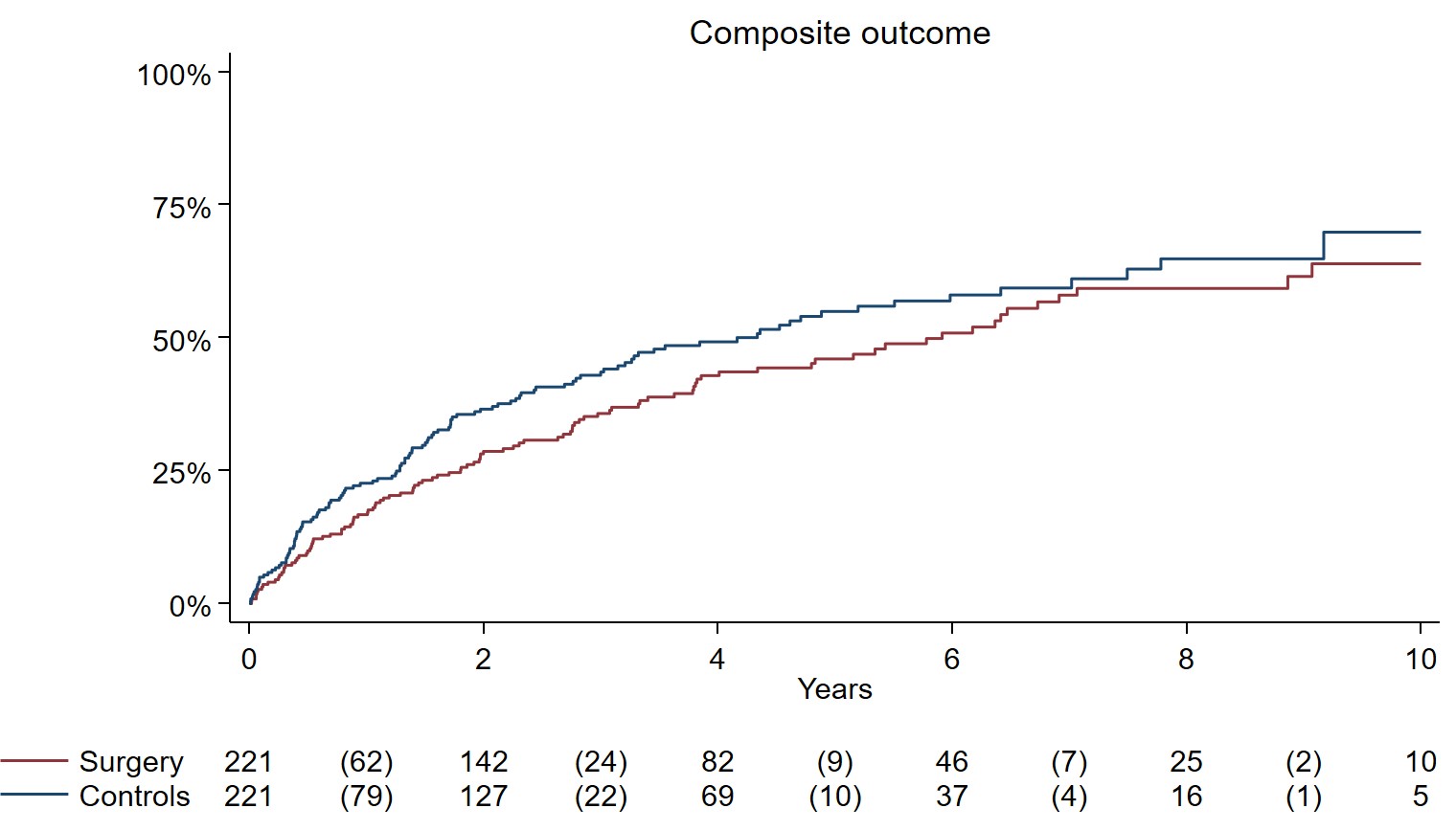
**

**Table S18.** Risk of IBD-related hospitalization, first systemic corticosteroid use, and first major IBD-related surgery in patients with bariatric surgery vs matched controls including BMI as a direct matching variable.

| **Outcome** | **N (%)** | | **N events** | | **Incidence rate (95% CI) per 100 PY** | | **Unadjusted HR***  **(95%CI)** | **Adjusted HR ****  **(95%CI)** |
| --- | --- | --- | --- | --- | --- | --- | --- | --- |
|  | **Bariatric surgery** | **Controls** | **Bariatric**  **surgery** | **Controls** | **Bariatric surgery** | **Controls** |  |  |
|  |  |  |  |  |  |  |  |  |
| Primary outcome |  |  |  |  |  |  |  |  |
| IBD subtype |  |  |  |  |  |  |  |  |
| IBD overall | 221 (100%) | 221 (100%) | 106 (48.0%) | 118 (53.4%) | 12.9 (10.4-15.3) | 16.8 (13.7-19.8) | 0.72 (0.57-0.91) | 0.72 (0.50-1.02) |
| CD | 80 (36.2%) | 80 (36.2%) | 47 (58.8%) | 48 (60.0%) | 18.4 (13.1-23.6) | 21.8 (15.6-27.9) | 0.77 (0.54-1.10) | 0.82 (0.46-1.47) |
| UC | 132 (59.7%) | 132 (59.7%) | 57 (43.2%) | 68 (51.5%) | 10.6 (7.8-13.3) | 14.9 (11.4-18.5) | 0.68 (0.50-0.92) | 0.67 (0.42-1.08) |
| IBD-U | 9 (4.1%) | 9 (4.1%) | 2 (22.2%) | 2 (22.2%) | 7.4 (0.0-17.6) | 7.4 (0.0-17.7) | 1.00 (0.25-4.00) | - |
| Type of surgery |  |  |  |  |  |  |  |  |
| Roux-en-Y gastric bypass | 91 (41.2%) | 91 (41.2%) | 47 (51.6%) | 62 (68.1%) | 11.3 (8.1-14.6) | 21.3 (16.0-26.6) | 0.53 (0.37-0.77) | 0.67 (0.37-1.23) |
| Sleeve gastrectomy | 130 (58.8%) | 130 (58.8%) | 59 (45.4%) | 56 (43.1%) | 14.4 (10.7-18.1) | 13.6 (10.0-17.1) | 0.91 (0.68-1.23) | 0.83 (0.53-1.32) |
| Secondary outcomes |  |  |  |  |  |  |  |  |
| *IBD-related hospitalization* |  |  |  |  |  |  |  |  |
| IBD overall | 221 (100%) | 221 (100%) | 16 (7.2%) | 20 (9.0%) | 1.3 (0.7-1.9) | 1.6 (0.9-2.3) | 0.84 (0.53-1.35) | 1.13 (0.53-2.42) |
| CD | 80 (36.2%) | 80 (36.2%) | 7 (8.8%) | 9 (11.3%) | 1.7 (0.4-2.9) | 2.2 (0.7-3.6) | 0.88 (0.43-1.80) | 1.81 (0.38-8.70) |
| UC | 132 (59.7%) | 132 (59.7%) | 8 (6.1%) | 11 (8.3%) | 1.0 (0.3-1.7) | 1.4 (0.6-2.2) | 0.73 (0.38-1.40) | 1.51 (0.31-7.49) |
| *Systemic corticosteroid use* |  |  |  |  |  |  |  |  |
| IBD overall | 221 (100%) | 221 (100%) | 83 (37.6%) | 89 (40.3%) | 9.0 (7.0-10.9) | 10.4 (8.3-12.6) | 0.90 (0.70-1.15) | 0.91 (0.63-1.33) |
| CD | 80 (36.2%) | 80 (36.2%) | 35 (43.8%) | 30 (37.5%) | 11.8 (7.9-15.6) | 10.0 (6.4-13.5) | 1.09 (0.72-1.64) | 1.55 (0.74-3.25) |
| UC | 132 (59.7%) | 132 (59.7%) | 47 (35.6%) | 57 (43.2%) | 7.8 (5.6-10.1) | 10.9 (8.0-13.7) | 0.82 (0.60-1.12) | 0.84 (0.52-1.36) |
| *Start of immunomodulator* |  |  |  |  |  |  |  |  |
| IBD overall | 221 (100%) | 221 (100%) | 39 (17.6%) | 48 (21.7%) | 3.5 (2.4-4.6) | 4.6 (3.3-5.9) | 0.83 (0.60-1.15) | 1.02 (0.60-1.75) |
| CD | 80 (36.2%) | 80 (36.2%) | 17 (21.3%) | 21 (26.3%) | 4.5 (2.4-6.7) | 6.2 (3.5-8.8) | 0.89 (0.55-1.43) | 1.40 (0.60-3.27) |
| UC | 132 (59.7%) | 132 (59.7%) | 21 (15.9%) | 27 (20.5%) | 3.0 (1.7-4.3) | 4.0 (2.5-5.5) | 0.74 (0.47-1.16) | 0.63 (0.28-1.41) |
| *Start of targeted therapy* |  |  |  |  |  |  |  |  |
| IBD overall | 221 (100%) | 221 (100%) | 21 (9.5%) | 32 (14.5%) | 1.7 (1.0-2.5) | 2.7 (1.8-3.6) | 0.63 (0.42-0.96) | 0.70 (0.36-1.36) |
| CD | 80 (36.2%) | 80 (36.2%) | 13 (16.3%) | 15 (18.8%) | 3.2 (1.5-5.0) | 3.9 (1.9-5.9) | 0.80 (0.47-1.37) | 0.93 (0.38-2.24) |
| UC | 132 (59.7%) | 132 (59.7%) | 8 (6.1%) | 17 (12.9%) | 1.0 (0.3-1.7) | 2.2 (1.2-3.3) | 0.47 (0.24-0.92) | 0.45 (0.15-1.40) |
| *Major IBD-related surgery* |  |  |  |  |  |  |  |  |
| IBD overall | 221 (100%) | 221 (100%) | 6 (2.7%) | 9 (4.1%) | 0.5 (0.1-0.8) | 0.7 (0.2-1.2) | 0.56 (0.25-1.24) | 1.00 (0.25-4.00) |
| CD | 80 (36.2%) | 80 (36.2%) | 5 (6.3%) | 6 (7.5%) | 1.2 (0.1-2.2) | 1.4 (0.3-2.5) | 0.67 (0.27-1.66) | 1.00 (0.20-4.95) |
| UC | 132 (59.7%) | 132 (59.7%) | 0 | 3 (2.3%) | 0 | 0 | - | - |

CD: Crohn’s disease; HR: hazard ratio; IBDU: Inflammatory bowel disease; IBD-U: Inflammatory bowel disease unclassified; IQR: Interquartile range; UC: Ulcerative colitis

*Conditioned on matching set;

**Conditioned on matching set and further adjusted immunomodulator before index date, ishemic heart disease, congestive heart failure, hypertension, and diabetes.

**References**

1. Keidar A, Hazan D, Sadot E, et al. The role of bariatric surgery in morbidly obese patients with inflammatory bowel disease. *Surg Obes Relat Dis* 2015;11(1):132-6. doi: 10.1016/j.soard.2014.06.022 [published Online First: 2014/12/31]

2. Aminian A, Andalib A, Ver MR, et al. Outcomes of Bariatric Surgery in Patients with Inflammatory Bowel Disease. *Obes Surg* 2016;26(6):1186-90. doi: 10.1007/s11695-015-1909-y [published Online First: 2015/10/01]

3. Sharma P, McCarty TR, Njei B. Impact of Bariatric Surgery on Outcomes of Patients with Inflammatory Bowel Disease: a Nationwide Inpatient Sample Analysis, 2004-2014. *Obes Surg* 2018;28(4):1015-24. doi: 10.1007/s11695-017-2959-0 [published Online First: 2017/10/20]

4. Aelfers S, Janssen IMC, Aarts EO, et al. Inflammatory Bowel Disease Is Not a Contraindication for Bariatric Surgery. *Obes Surg* 2018;28(6):1681-87. doi: 10.1007/s11695-017-3076-9 [published Online First: 2017/12/29]

5. Hudson JL, Barnes EL, Herfarth HH, et al. Bariatric Surgery Is a Safe and Effective Option for Patients with Inflammatory Bowel Diseases: A Case Series and Systematic Review of the Literature. *Inflamm Intest Dis* 2019;3(4):173-79. doi: 10.1159/000496925 [published Online First: 2019/05/22]

6. Heshmati K, Lo T, Tavakkoli A, et al. Short-Term Outcomes of Inflammatory Bowel Disease after Roux-en-Y Gastric Bypass vs Sleeve Gastrectomy. *J Am Coll Surg* 2019;228(6):893-901.e1. doi: 10.1016/j.jamcollsurg.2019.01.021 [published Online First: 2019/02/24]

7. McKenna NP, Habermann EB, Sada A, et al. Is Bariatric Surgery Safe and Effective in Patients with Inflammatory Bowel Disease? *Obes Surg* 2020;30(3):882-88. doi: 10.1007/s11695-019-04267-8 [published Online First: 2019/11/24]

8. Braga Neto MB, Gregory MH, Ramos GP, et al. Impact of Bariatric Surgery on the Long-term Disease Course of Inflammatory Bowel Disease. *Inflamm Bowel Dis* 2020;26(7):1089-97. doi: 10.1093/ibd/izz236 [published Online First: 2019/10/16]

9. Reenaers C, de Roover A, Kohnen L, et al. Bariatric Surgery in Patients With Inflammatory Bowel Disease: A Case-Control Study from the GETAID. *Inflamm Bowel Dis* 2022;28(8):1198-206. doi: 10.1093/ibd/izab249 [published Online First: 2021/10/13]

10. Corbière L, Scanff A, Desfourneaux V, et al. Outcomes of bariatric surgery in patients with inflammatory bowel disease from a French nationwide database. *Br J Surg* 2023;110(2):251-59. doi: 10.1093/bjs/znac398 [published Online First: 2022/12/01]

11. Desai A, Hashash JG, Baker G, et al. Effect of Bariatric Surgery on Disease Outcomes in Patients with Inflammatory Bowel Disease: A US-based Propensity Matched Cohort Study. *J Clin Gastroenterol* 2024.58(5):447-453

12. Ludvigsson JF, Almqvist C, Bonamy AE, et al. Registers of the Swedish total population and their use in medical research. *Eur J Epidemiol* 2016;31(2):125-36. doi: 10.1007/s10654-016-0117-y [published Online First: 2016/01/16]

13. Ludvigsson JF, Svedberg P, Olen O, et al. The longitudinal integrated database for health insurance and labour market studies (LISA) and its use in medical research. *Eur J Epidemiol* 2019;34(4):423-37. doi: 10.1007/s10654-019-00511-8 [published Online First: 2019/04/01]

14. Ludvigsson JF, Andersson E, Ekbom A, et al. External review and validation of the Swedish national inpatient register. *BMC Public Health* 2011;11(1):450. doi: 1471-2458-11-450 [pii]

10.1186/1471-2458-11-450 [published Online First: 2011/06/11]

15. Wettermark B, Hammar N Fau - Fored CM, Fored Cm Fau - Leimanis A, et al. The new Swedish Prescribed Drug Register--opportunities for pharmacoepidemiological research and experience from the first six months. (1053-8569 (Print))

16. Ludvigsson JF, Lashkariani M. Cohort profile: ESPRESSO (Epidemiology Strengthened by histoPathology Reports in Sweden). *Clin Epidemiol* 2019;11:101-14. doi: 10.2147/clep.S191914 [published Online First: 20190114]

17. Barlow L, Westergren K, Holmberg L, et al. The completeness of the Swedish Cancer Register: a sample survey for year 1998. *Acta Oncol* 2009;48(1):27-33. doi: 10.1080/02841860802247664 [published Online First: 2008/09/04]

18. Brooke HL, Talback M, Hornblad J, et al. The Swedish cause of death register. *European journal of epidemiology* 2017;32(9):765-73. doi: 10.1007/s10654-017-0316-1 [published Online First: 20171005]

19. Ludvigsson JF, Andersson M, Bengtsson J, et al. Swedish Inflammatory Bowel Disease Register (SWIBREG) - a nationwide quality register. *Scand J Gastroenterol* 2019;54(9):1089-101. doi: 10.1080/00365521.2019.1660799 [published Online First: 20190909]

20. Sundbom M, Naslund I, Naslund E, et al. High acquisition rate and internal validity in the Scandinavian Obesity Surgery Registry. *Surgery for obesity and related diseases : official journal of the American Society for Bariatric Surgery* 2021;17(3):606-14. doi: <https://dx.doi.org/10.1016/j.soard.2020.10.017>

21. Everhov AH, Olen O, Ludvigsson JF. Editorial: importance of definition of inflammatory bowel disease and an increased incidence in children. *Aliment Pharmacol Ther* 2017;45(10):1369-70. doi: 10.1111/apt.14035

22. Lophaven SN, Lynge E, Burisch J. The incidence of inflammatory bowel disease in Denmark 1980-2013: a nationwide cohort study. *Aliment Pharmacol Ther* 2017;45(7):961-72. doi: 10.1111/apt.13971

23. Jakobsson GL, Sternegård E, Olén O, et al. Validating inflammatory bowel disease (IBD) in the Swedish National Patient Register and the Swedish Quality Register for IBD (SWIBREG). *Scand J Gastroenterol* 2017;52(2):216-21. doi: 10.1080/00365521.2016.1246605 [published Online First: 20161031]

24. Nguyen LH, Ortqvist AK, Cao Y, et al. Antibiotic use and the development of inflammatory bowel disease: a national case-control study in Sweden. *The lancet Gastroenterology & hepatology* 2020;5(11):986-95. doi: 10.1016/S2468-1253(20)30267-3 [published Online First: 20200817]

25. Albaek Jacobsen H, Jess T, Larsen L. Validity of Inflammatory Bowel Disease Diagnoses in the Danish National Patient Registry: A Population-Based Study from the North Denmark Region. *Clinical epidemiology* 2022;14:1099-109. doi: 10.2147/CLEP.S378003 [published Online First: 2022/10/14]

26. Mouratidou N, Malmborg P, Jaras J, et al. Identification of Childhood-Onset Inflammatory Bowel Disease in Swedish Healthcare Registers: A Validation Study. *Clinical epidemiology* 2022;14:591-600. doi: 10.2147/CLEP.S358031 [published Online First: 2022/05/07]

27. Everhov AH, Sachs MC, Malmborg P, et al. Changes in inflammatory bowel disease subtype during follow-up and over time in 44,302 patients. *Scandinavian journal of gastroenterology* 2019;54(1):55-63. doi: 10.1080/00365521.2018.1564361 [published Online First: 20190131]

28. Satsangi J, Silverberg MS, Vermeire S, et al. The Montreal classification of inflammatory bowel disease: controversies, consensus, and implications. *Gut* 2006;55(6):749-53. doi: 10.1136/gut.2005.082909 [published Online First: 2006/05/16]

29. Shrestha S, Olen O, Eriksson C, et al. The use of ICD codes to identify IBD subtypes and phenotypes of the Montreal classification in the Swedish National Patient Register. *Scandinavian journal of gastroenterology* 2020;55(4):430-35. doi: 10.1080/00365521.2020.1740778 [published Online First: 20200506]

30. Forss A, Myrelid P, Olen O, et al. Validating surgical procedure codes for inflammatory bowel disease in the Swedish National Patient Register. *BMC medical informatics and decision making* 2019;19(1):217. doi: 10.1186/s12911-019-0948-z [published Online First: 20191111]

1. [↑](#footnote-ref-1)
